# Supplementary material for: The MAST kinase KIN-4 carries out mitotic entry functions of Greatwall in C. elegans
Source: EMBO J. 2025 Feb 17;44(7):1943–74. doi: 10.1038/s44318-025-00364-w (PMC11961639; doi:10.1038/s44318-025-00364-w)
Supplement: Supplementary file 1 — Table EV1 [file 44318_2025_364_MOESM1_ESM.docx]

**Table EV1: ENSA-1 is phosphorylated at multiple sites in *C. elegans* embryos**

Phosphopeptides matching the Cyclin-Cdk consensus are highlighted in yellow, and those matching the DSG sequence motif are highlighted in orange.
