# Supplementary material for: The MAST kinase KIN-4 carries out mitotic entry functions of Greatwall in C. elegans
Source: EMBO J. 2025 Feb 17;44(7):1943–74. doi: 10.1038/s44318-025-00364-w (PMC11961639; doi:10.1038/s44318-025-00364-w)
Supplement: Supplementary file 2 — Table EV2 [file 44318_2025_364_MOESM2_ESM.docx]

**Table EV2: List of *C. elegans* strains used in this study:**

| **Name/alias** | **Description** | **Genotype** | **Source** |
| --- | --- | --- | --- |
| N2 |  | *C. elegans* N2 Bristol | CGC |
| MT2124 | *let-60(gf)* | *let-60(ne1046)* | CGC |
| WLP1241/UP994 | *sur-6* null | *sur-6(sv30) I/hT2 [bli-4(e937) let-?(q782) qIs48] (I;III).* | CGC |
| WLP726/EU1062 | *sur-6*ts | *sur-6(or550)* I | CGC |
| WLP1039/MGL1 | *ensa-1 DSG∆* | *ensa-1(tm2810)* I | This study |
| WLP1273 | *ensa-1 DSG∆/sur6*ts | *ensa-1(tm2810) I; sur-6 (lea1)*ts I | This study |
| WLP1274 | *ensa-1 DSG∆/sur6*ts | *ensa-1 (tm2810) I; sur-6 (lea1) ts I* | This study |
| WLP1100/ MCP399 | *ensa-1 S61A* | *ensa-1(bab399)[Ensa-1(S61A)]* | This study |
| WLP1264 | *ensa-1 S61A, sur6*ts | *ensa-1(bab399)[Ensa-1(S61A)] I; sur-6 ts I* | This study |
| WLP1265 | *ensa-1 S61A, sur6*ts | *ensa-1(bab399)[Ensa-1(S61A)] I; sur-6 ts I* | This study |
| WLP1085/ FX01049 | *kin-4∆* | *kin-4(tm1049)* | Shohei Mitani |
| WLP1207 | *sur6*ts*, kin-4∆* | *sur-6(or550) I x kin-4 tm1049 IV* | This study |
| WLP1208 | *sur6*ts*, kin-4∆* | *sur-6(or550) I x kin-4 tm1049 IV* | This study |
| WLP1275 | *sur6*ts | *sur-6(lea1)* ts I | This study |
| WLP1226/JDU233 | *H2B::mCherry;α-tubulin::GFP II* | *ijmSi63 [pJD520; mosII_5'mex-5_GFP::tba-2; mCherry::his-11; cb-unc-119(+)] II; unc-119(ed3) III* | Julien Dumont |
| WLP1237 | *H2B::mCherry;α-tubulin::GFPII, sur6*ts | *sur-6(or550)* I ; ijmSi63 [pJD520; mosII_5'mex-5_GFP::tba-2; mCherry::his-11; cb-unc-119(+)] II; unc-119(ed3) III | This study |
