# Supplementary figures and images for: The MAST kinase KIN-4 carries out mitotic entry functions of Greatwall in C. elegans
Source: EMBO J. 2025 Feb 17;44(7):1943–74. doi: 10.1038/s44318-025-00364-w (PMC11961639; doi:10.1038/s44318-025-00364-w)

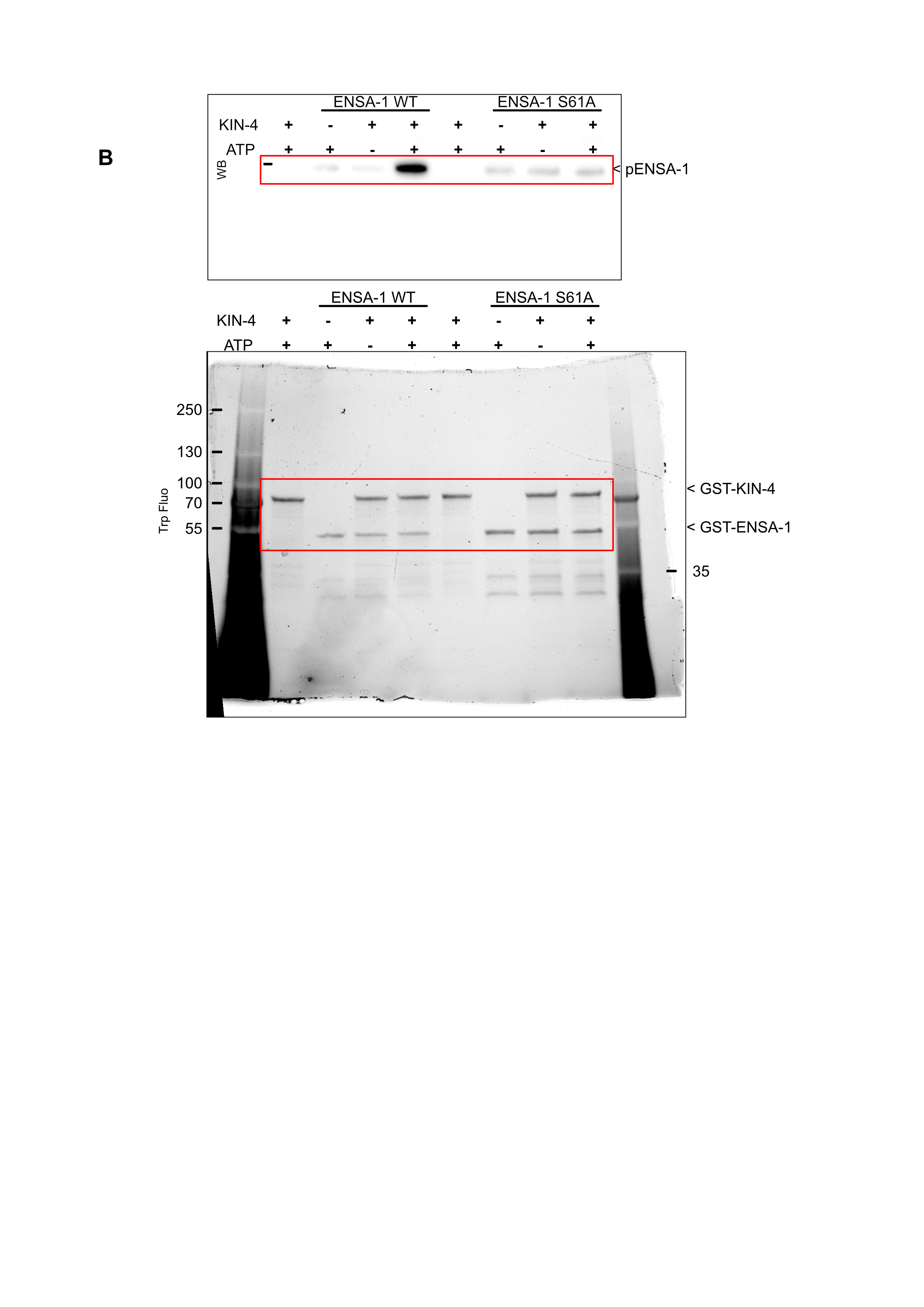

Supplement: Supplementary file 9 — Source data Fig. 6 [file 44318_2025_364_MOESM9_ESM.zip › Figure 6/6B/EMBOJ-2024-117214R_SourceDataForFigure6B.tiff]

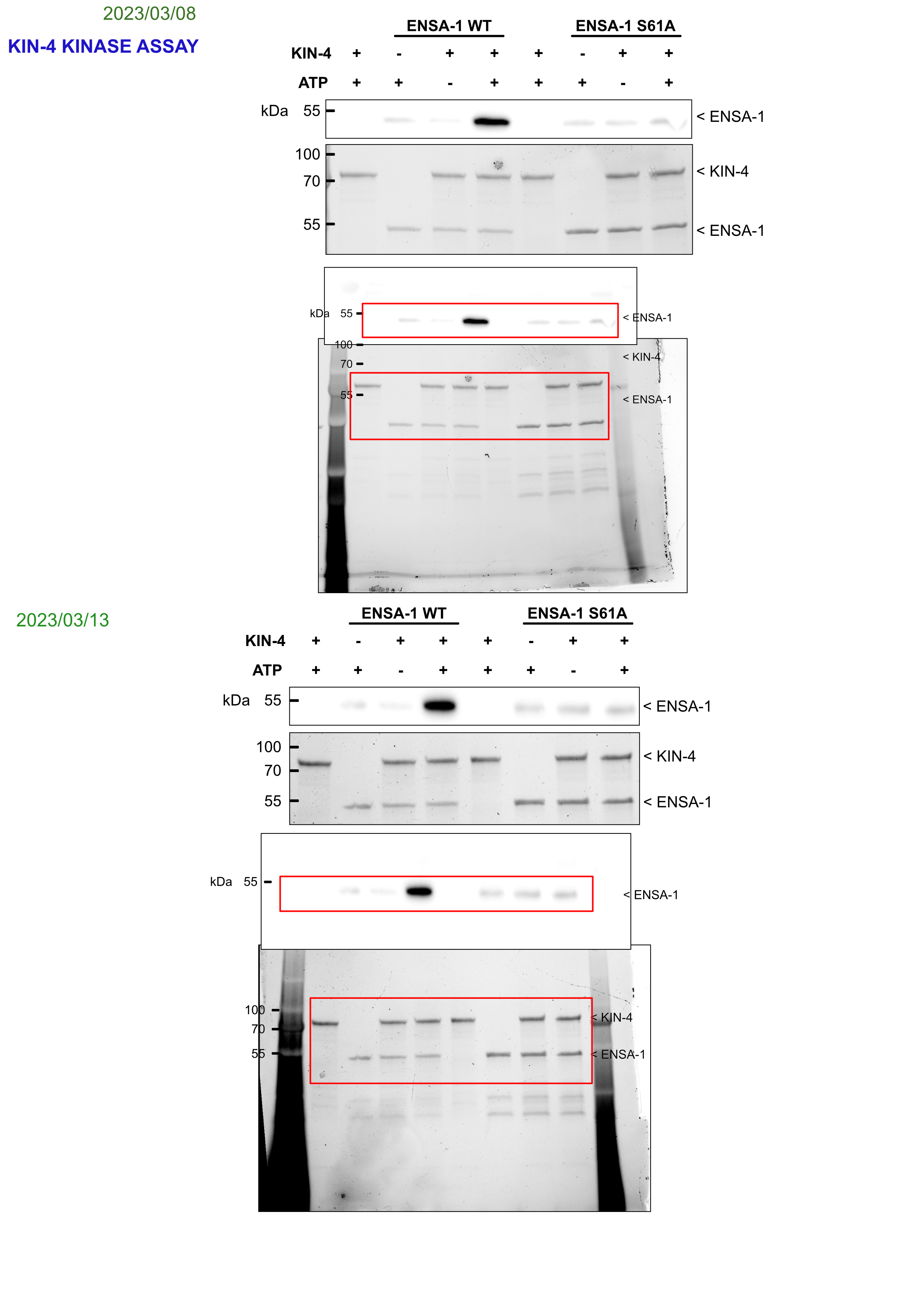

Supplement: Supplementary file 9 — Source data Fig. 6 [file 44318_2025_364_MOESM9_ESM.zip › Figure 6/6B/Replicates/F 6.B BLOT REPLICATES.tiff]

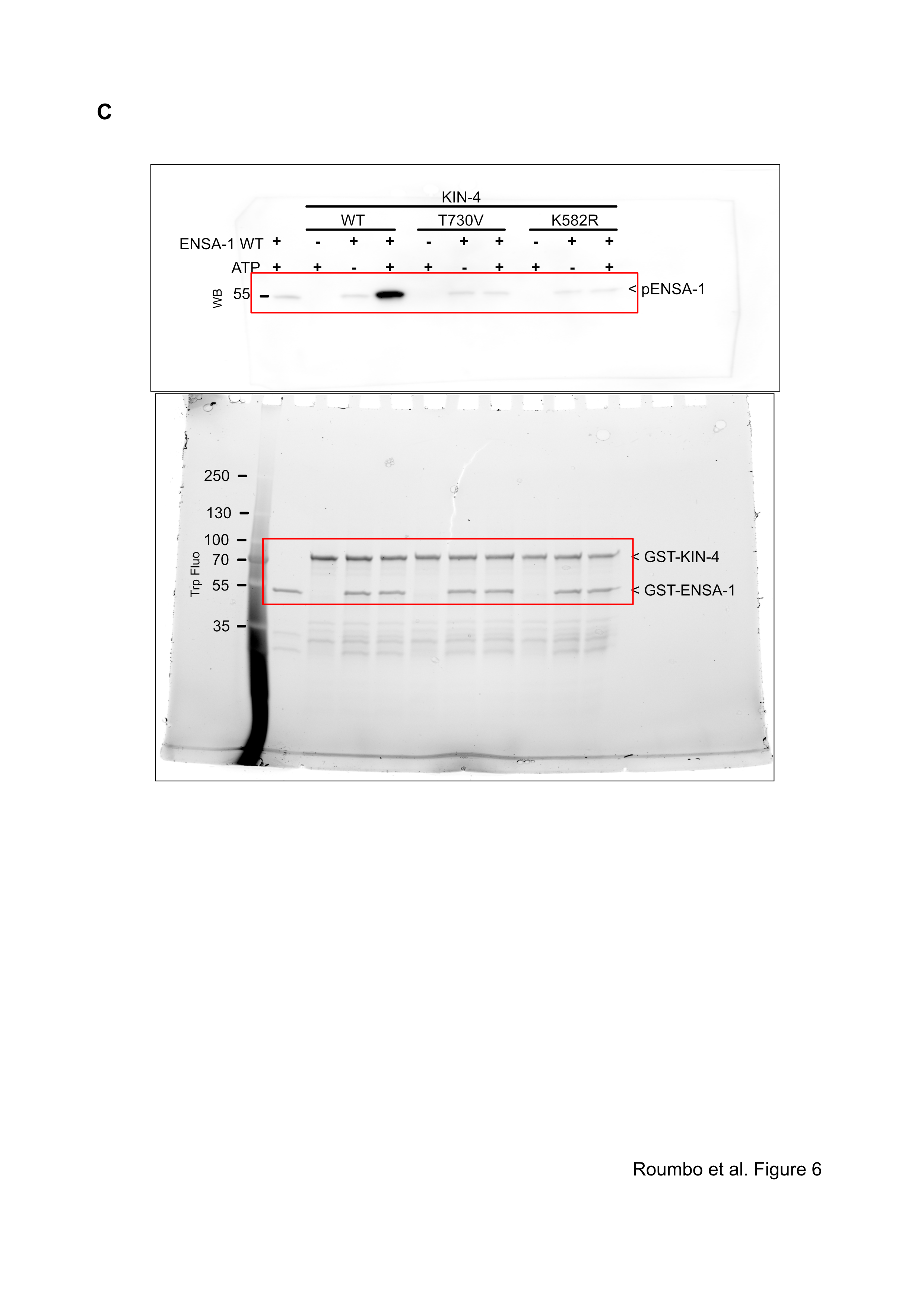

Supplement: Supplementary file 9 — Source data Fig. 6 [file 44318_2025_364_MOESM9_ESM.zip › Figure 6/6C/EMBOJ-2024-117214R_SourceDataForFigure6C.tiff]

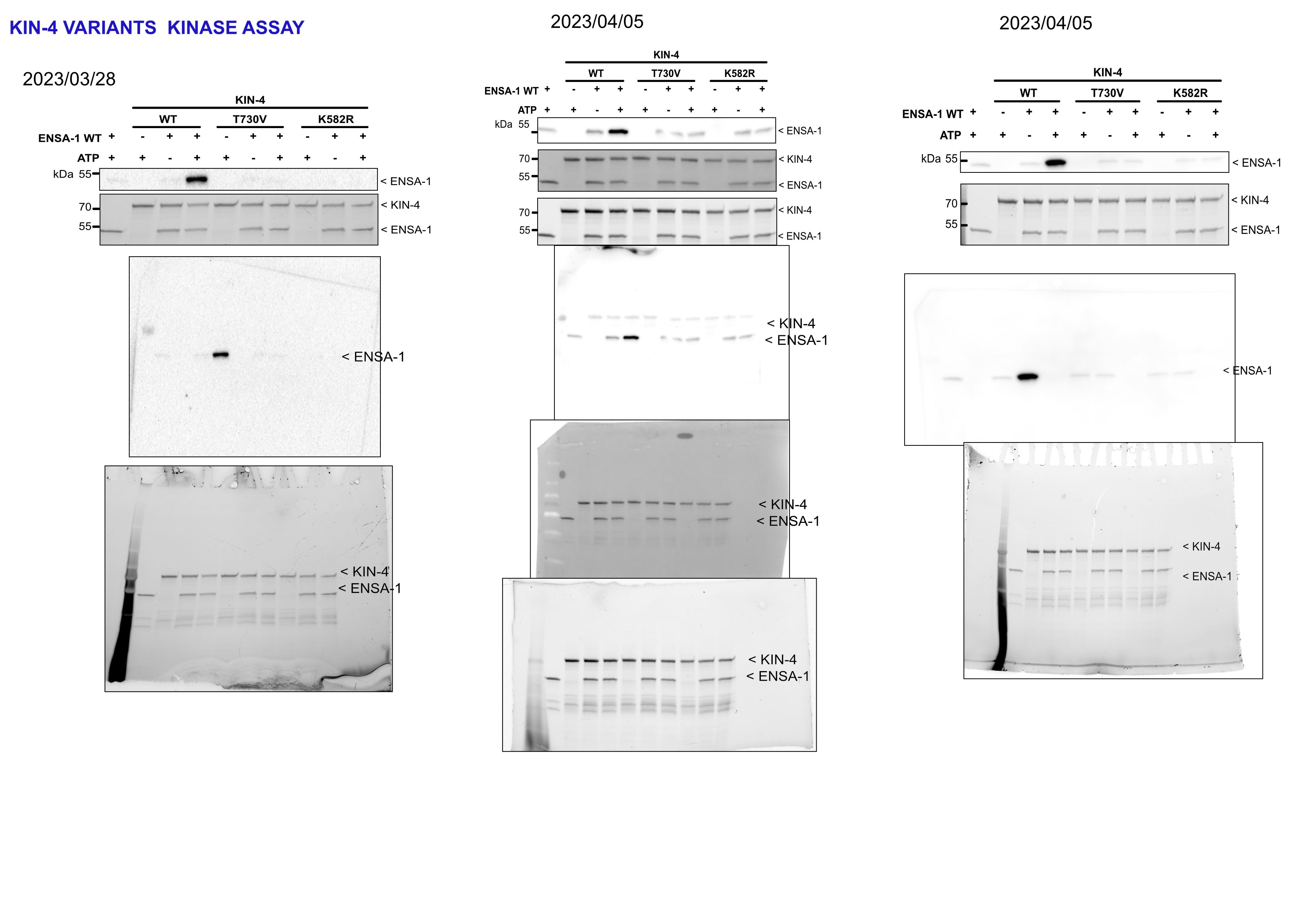

Supplement: Supplementary file 9 — Source data Fig. 6 [file 44318_2025_364_MOESM9_ESM.zip › Figure 6/6C/replicates/F 6 C KIN-4 Variants replicates.tiff]

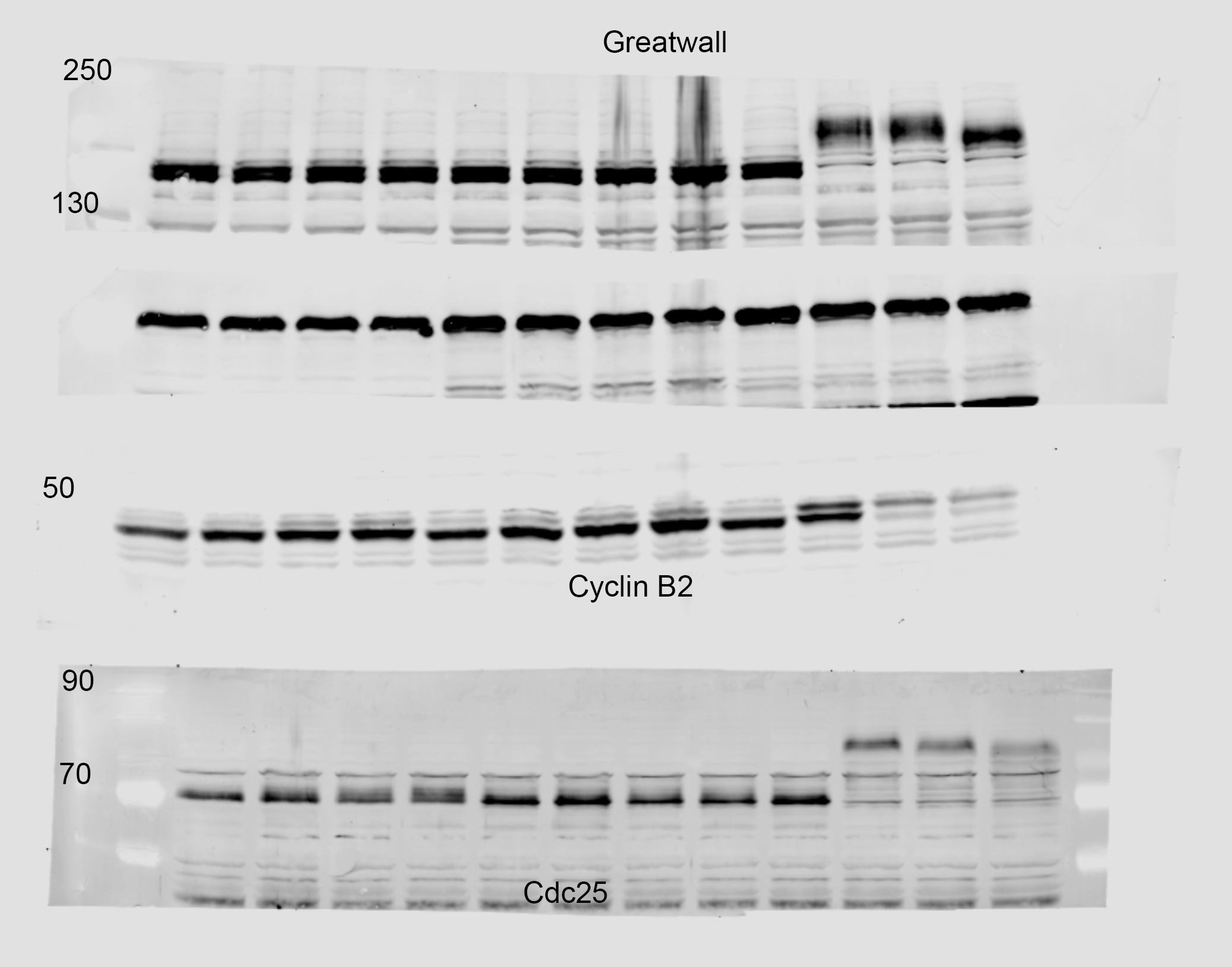

Supplement: Supplementary file 10 — Source data Fig. 7 [file 44318_2025_364_MOESM10_ESM.zip › Figure 7/7A/Inter et kin4 en gris.tif]

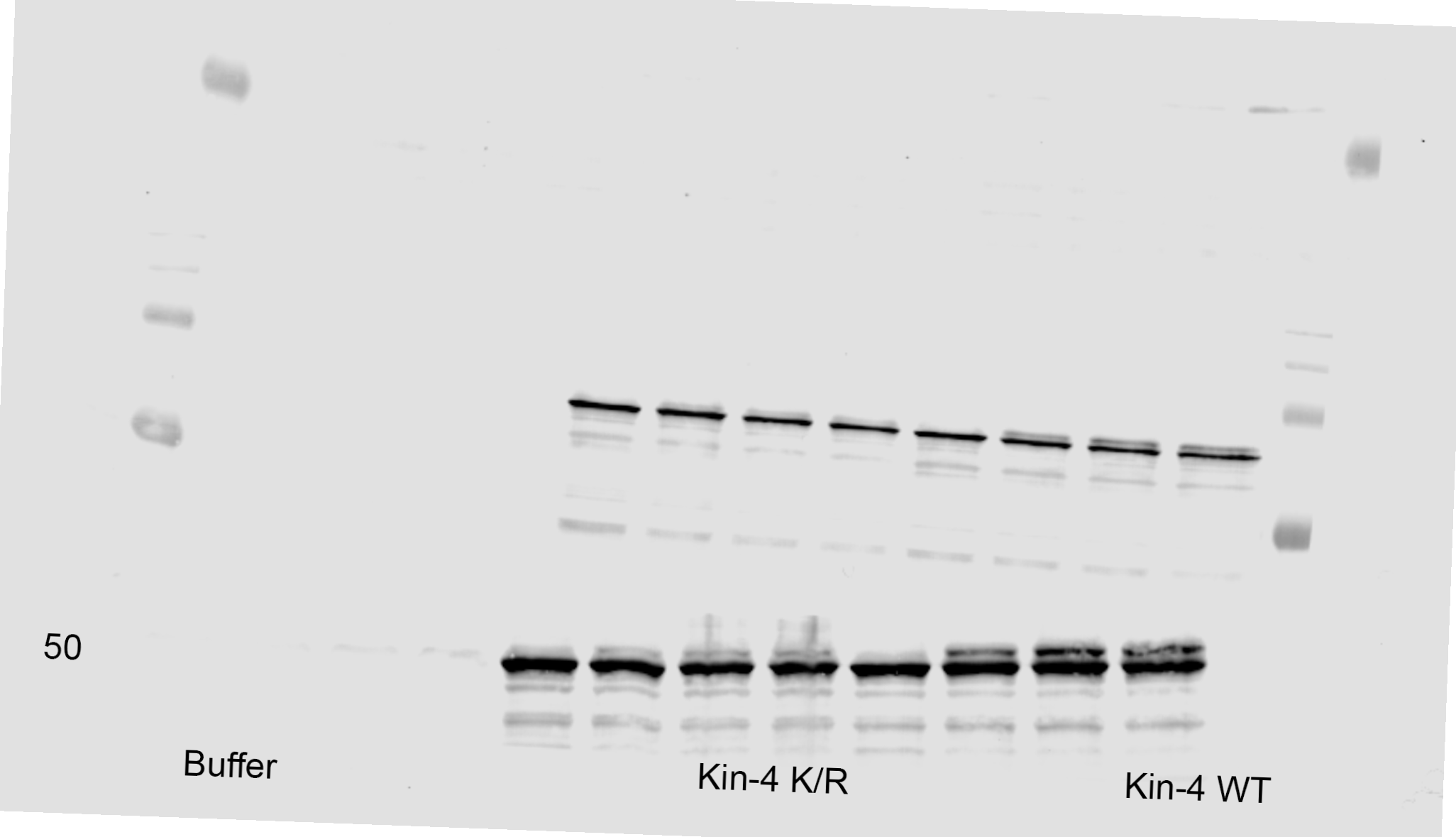

Supplement: Supplementary file 10 — Source data Fig. 7 [file 44318_2025_364_MOESM10_ESM.zip › Figure 7/7A/Inter et Kin4 Gris Gst pour kin4 rotated.tif]

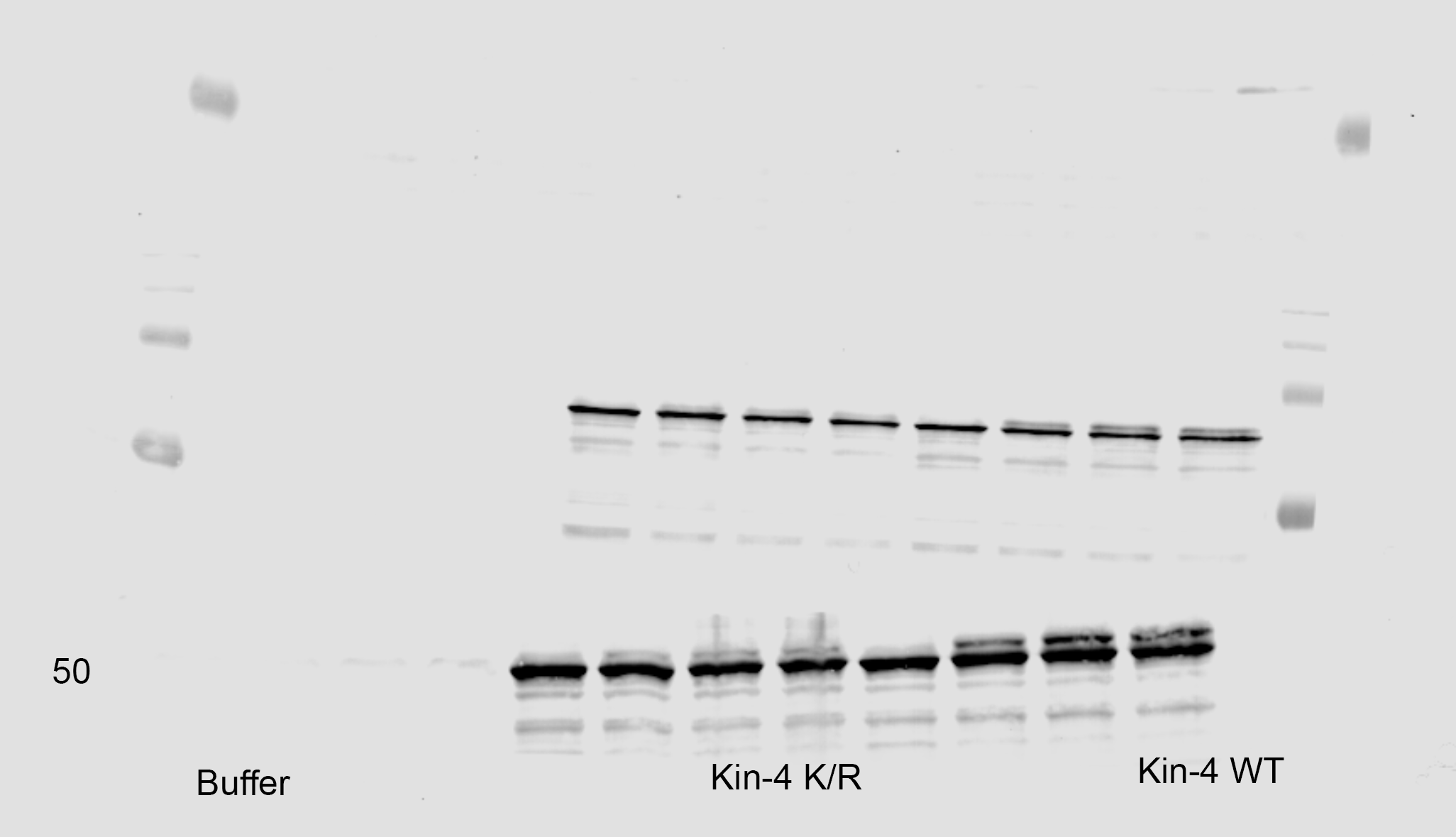

Supplement: Supplementary file 10 — Source data Fig. 7 [file 44318_2025_364_MOESM10_ESM.zip › Figure 7/7A/Inter et Kin4 Gris Gst pour kin4.tif]

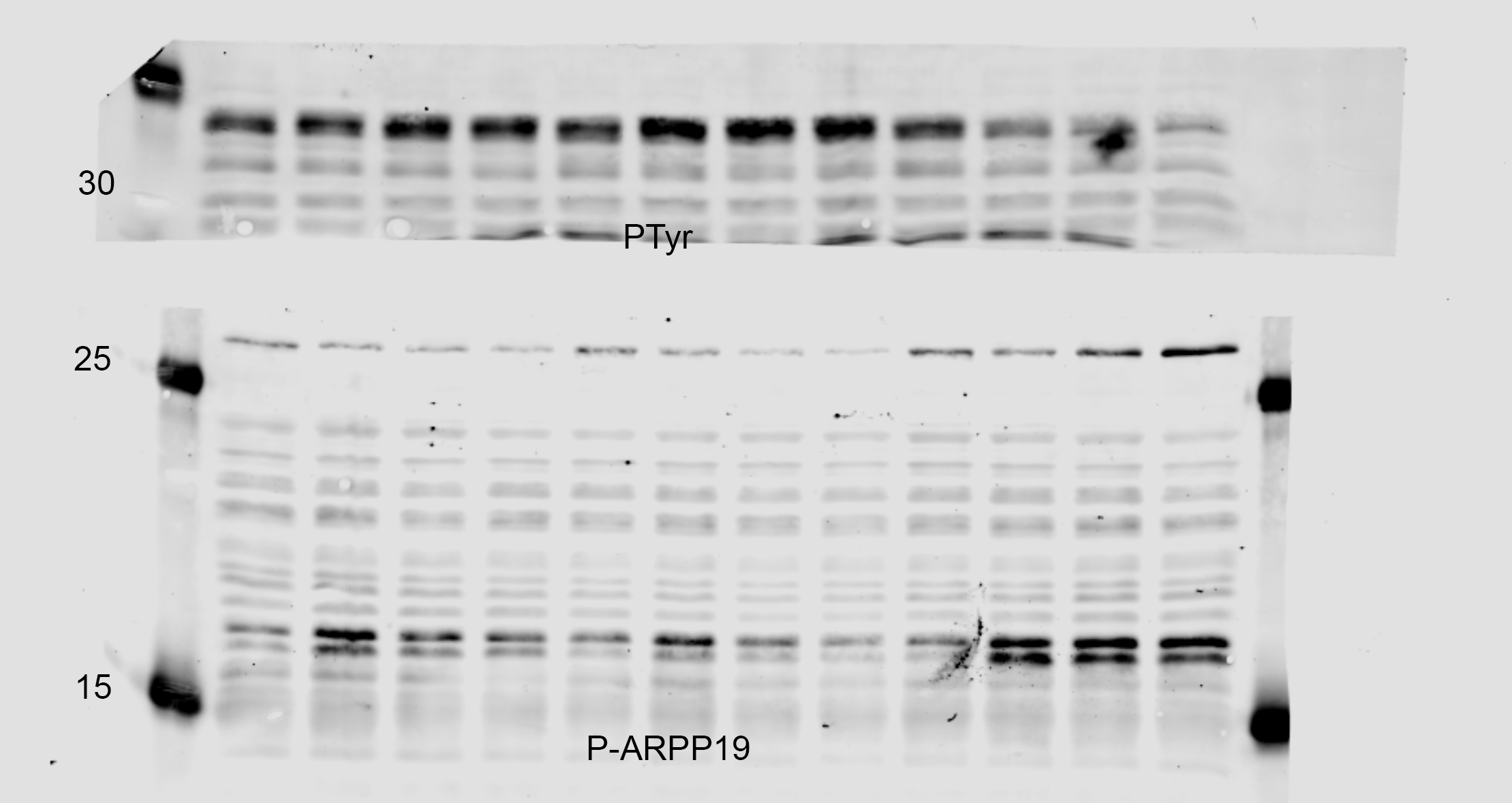

Supplement: Supplementary file 10 — Source data Fig. 7 [file 44318_2025_364_MOESM10_ESM.zip › Figure 7/7A/Inter et Kin4 Gris Pyr et P-Arpp19.tif]

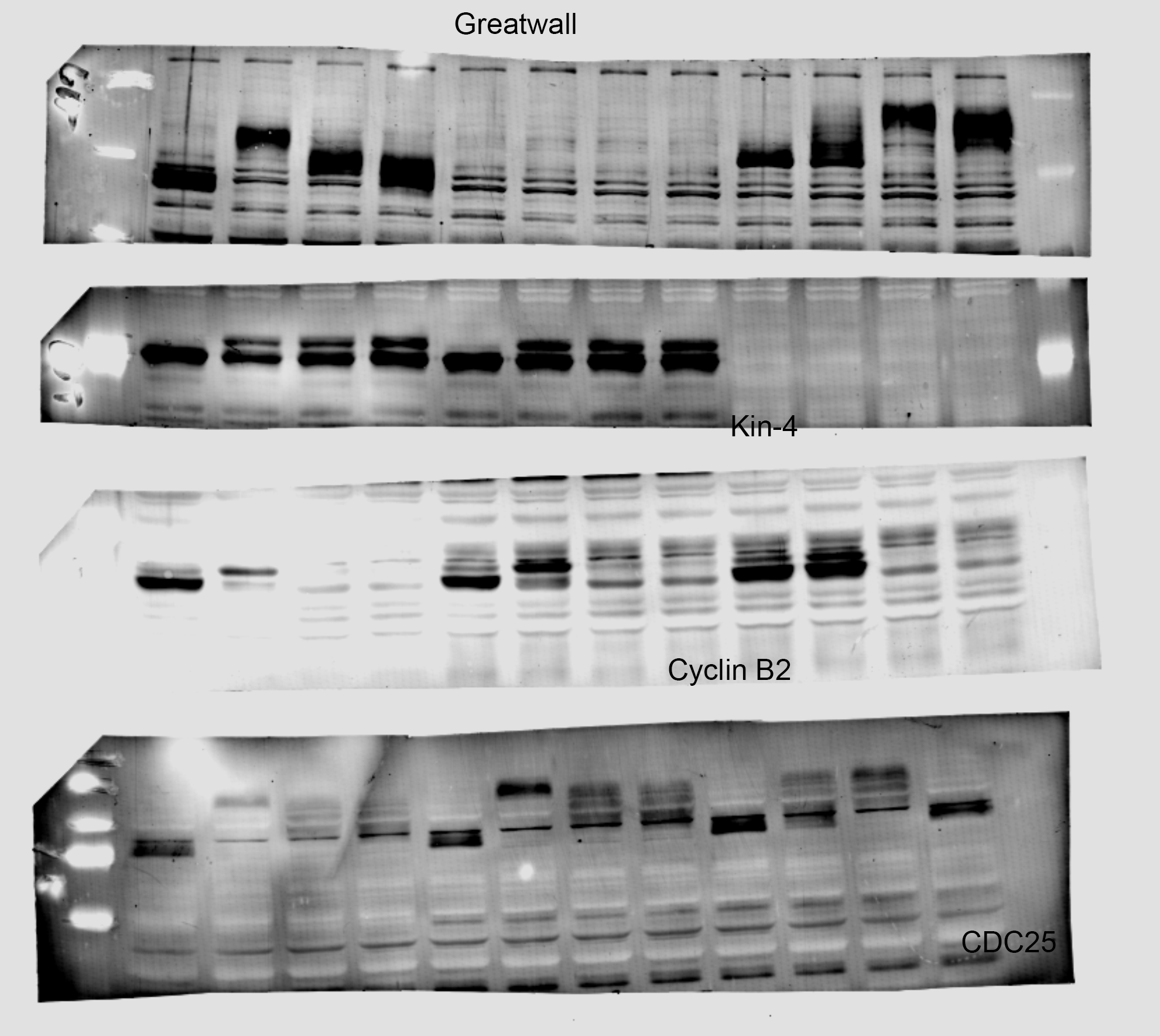

Supplement: Supplementary file 10 — Source data Fig. 7 [file 44318_2025_364_MOESM10_ESM.zip › Figure 7/7B/Inter delta Gwl et Kin4 cdc25 - Copie.tif]

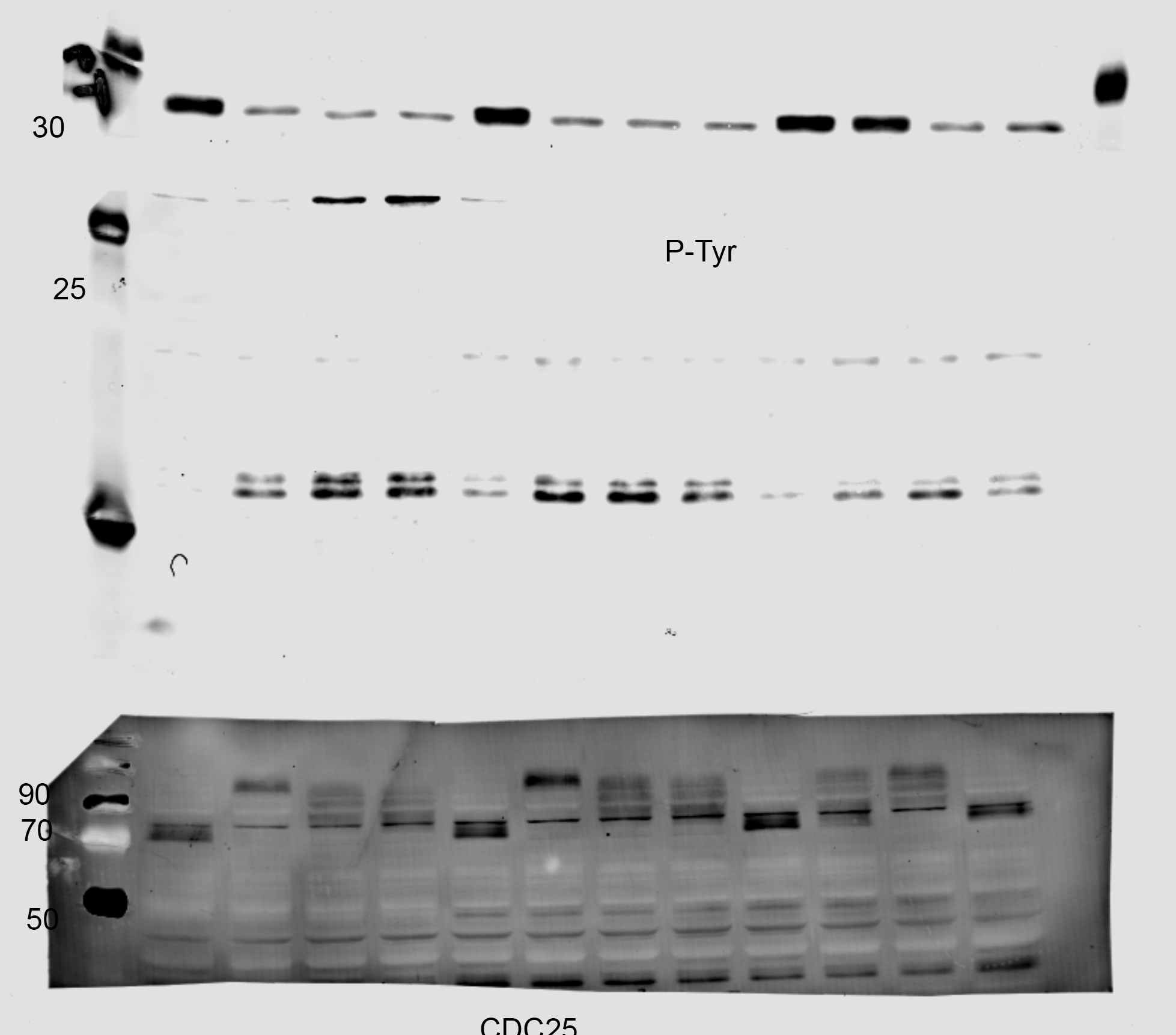

Supplement: Supplementary file 10 — Source data Fig. 7 [file 44318_2025_364_MOESM10_ESM.zip › Figure 7/7B/Inter delta Gwl et Kin4 cdc25 et Ptyr.tif]

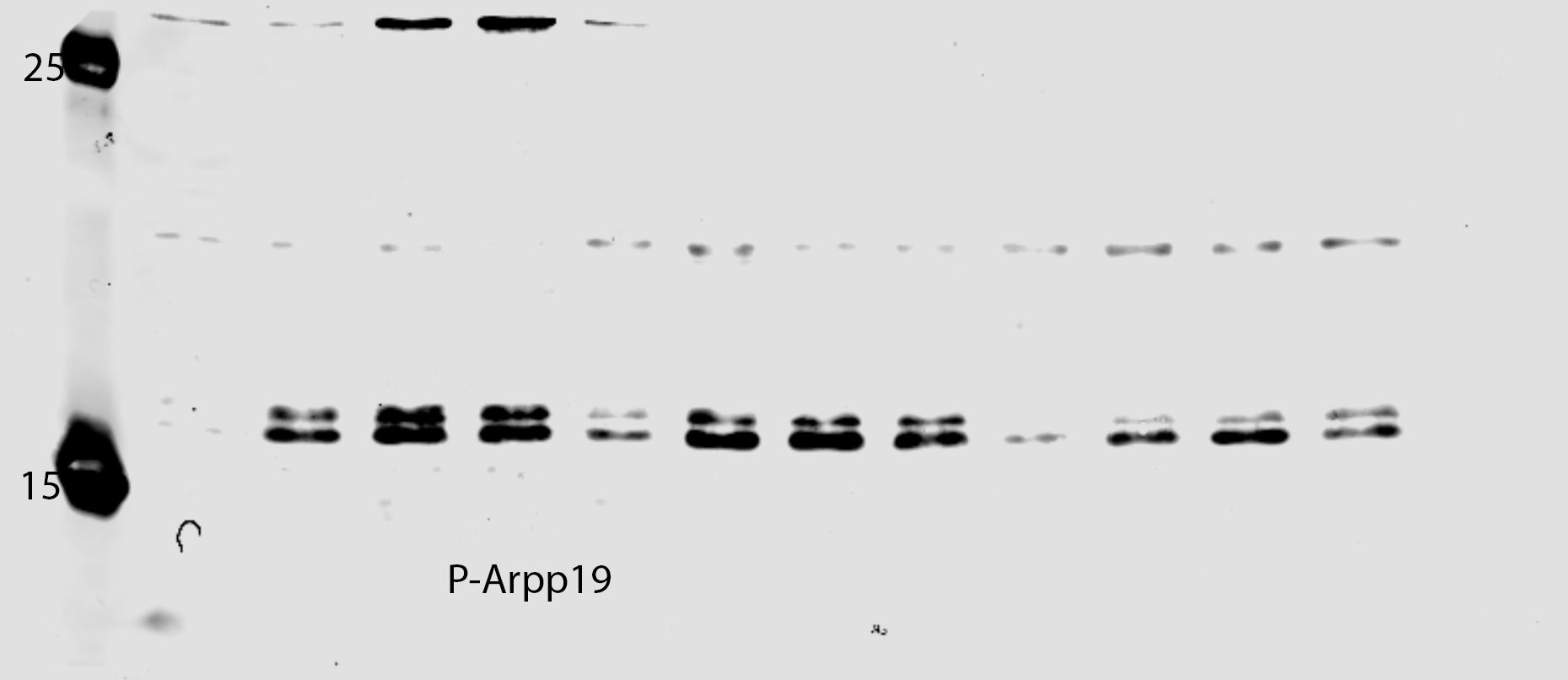

Supplement: Supplementary file 10 — Source data Fig. 7 [file 44318_2025_364_MOESM10_ESM.zip › Figure 7/7B/Inter delta Gwl et Kin4 P-Arpp19.tif]

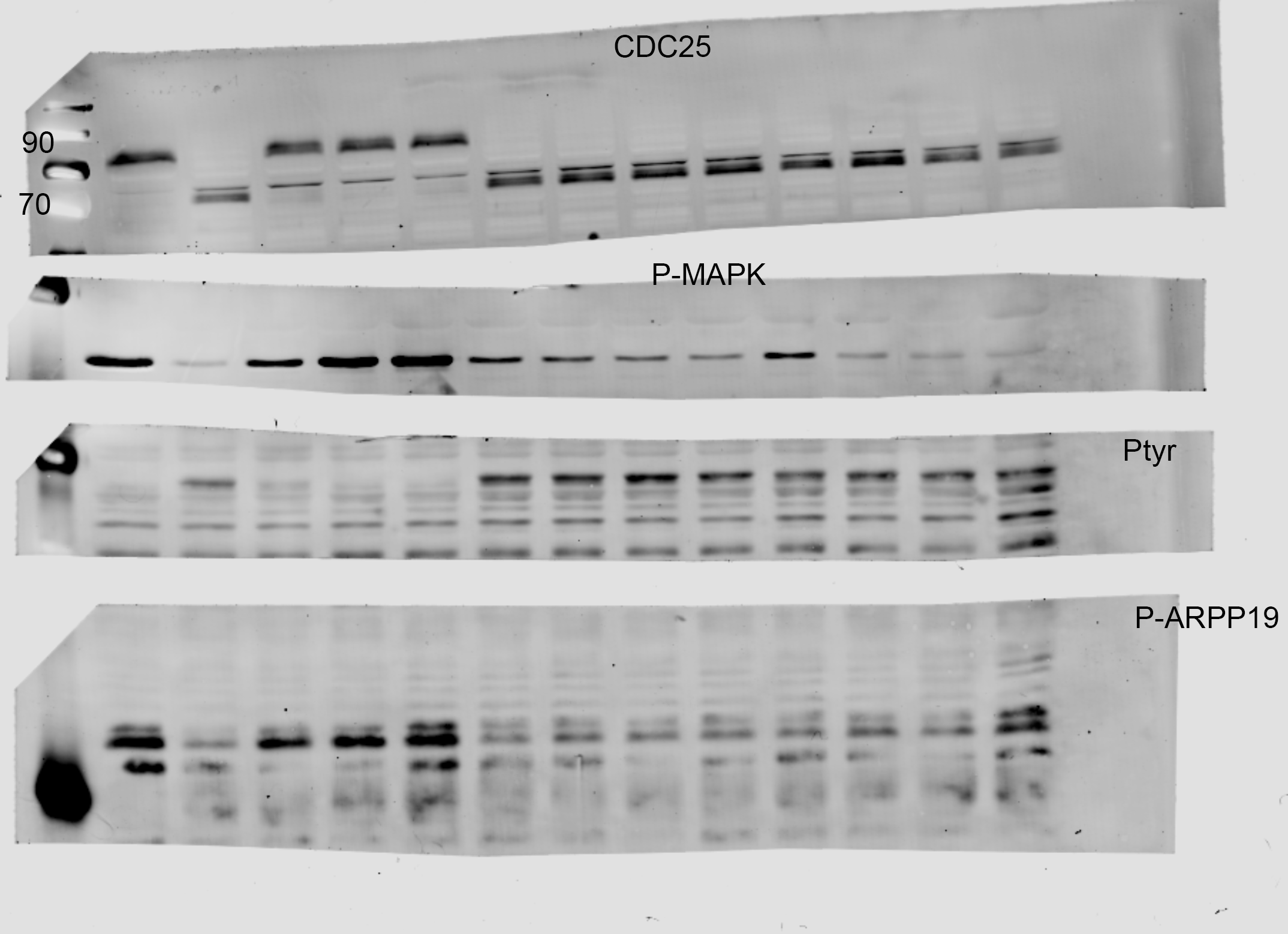

Supplement: Supplementary file 10 — Source data Fig. 7 [file 44318_2025_364_MOESM10_ESM.zip › Figure 7/7C/CSF delta Gwl et Kin4 cdc25.tif]

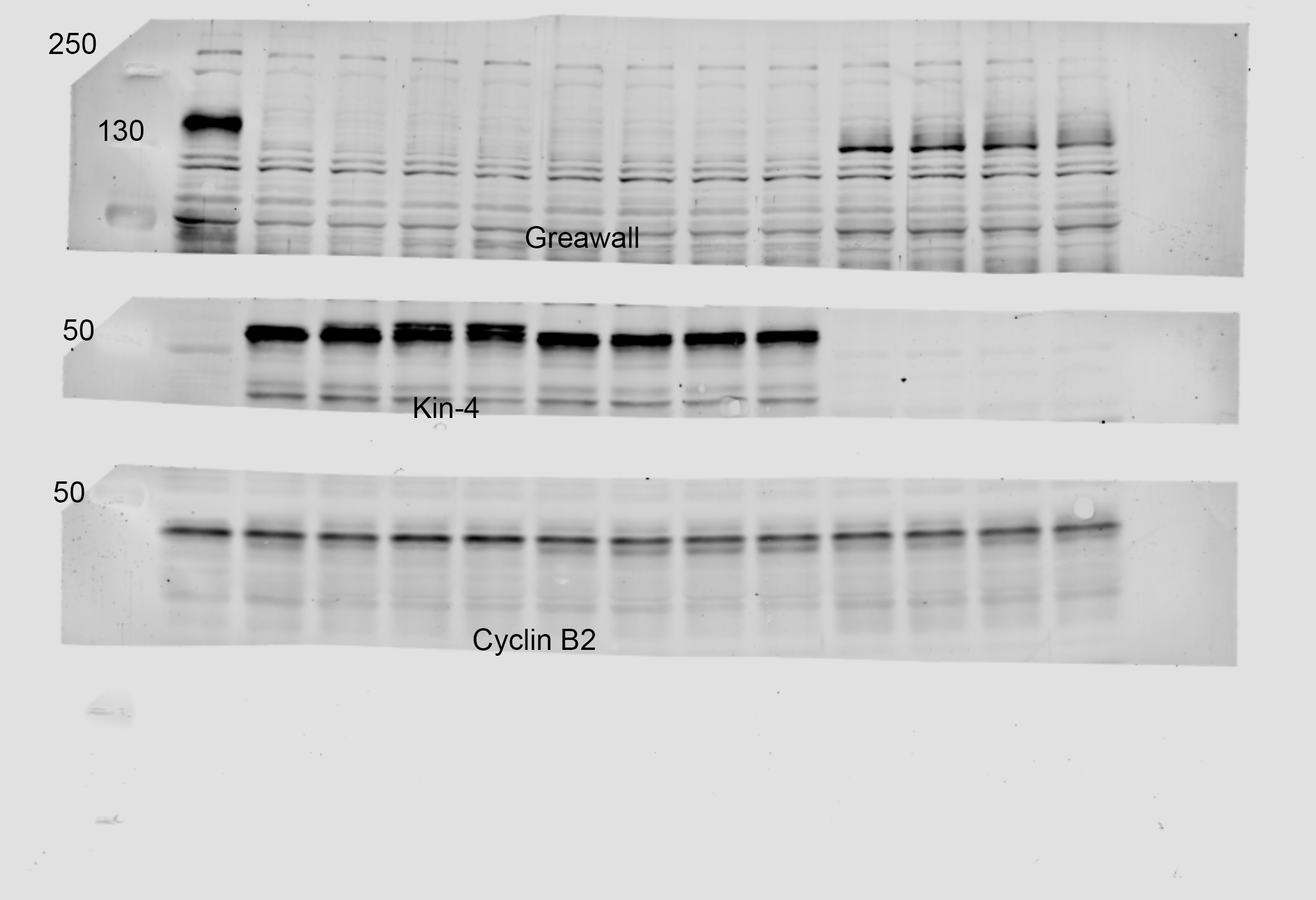

Supplement: Supplementary file 10 — Source data Fig. 7 [file 44318_2025_364_MOESM10_ESM.zip › Figure 7/7C/CSF delta Gwl et Kin4 Gwl Gst et B2.tif]

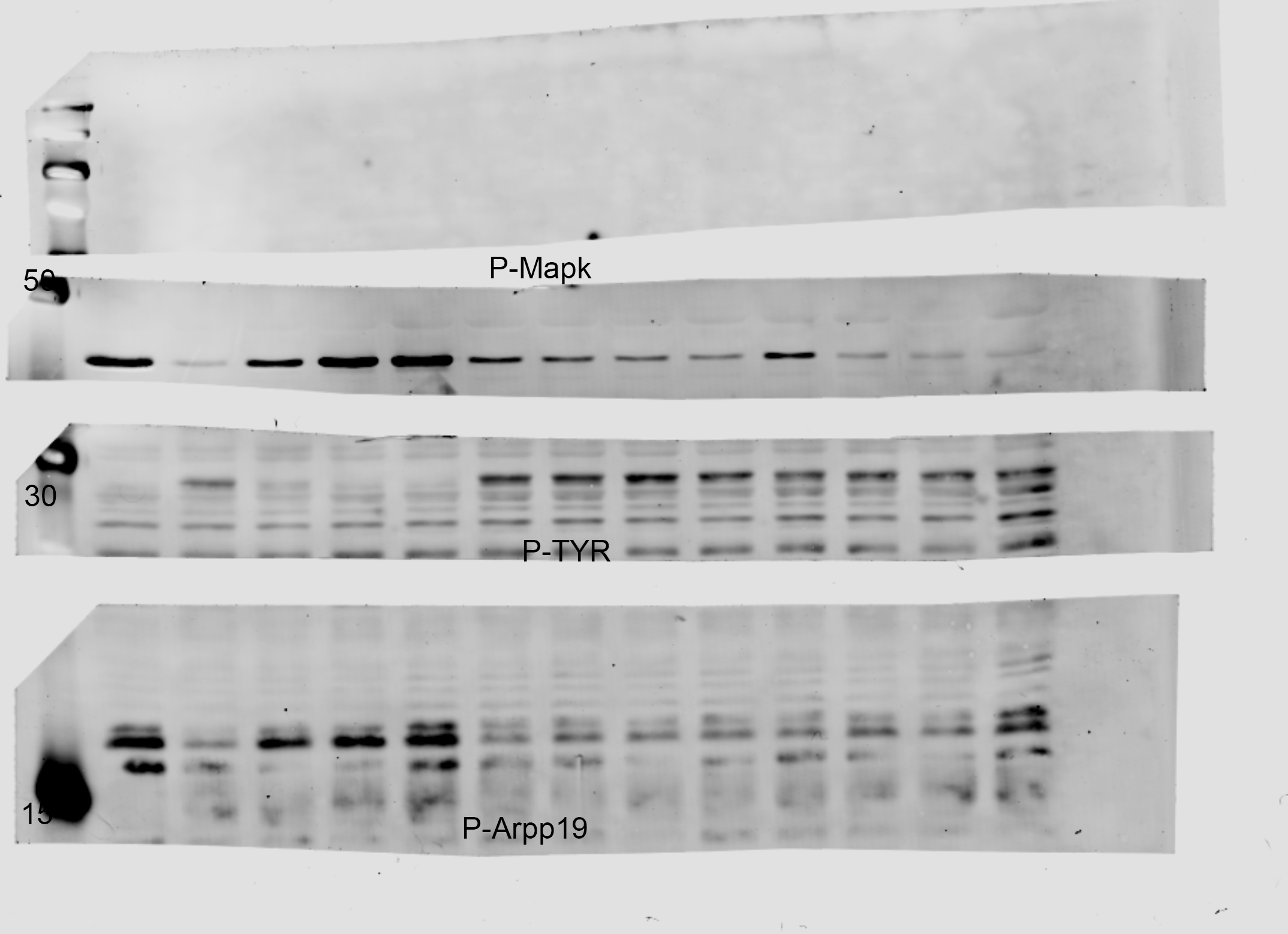

Supplement: Supplementary file 10 — Source data Fig. 7 [file 44318_2025_364_MOESM10_ESM.zip › Figure 7/7C/CSF delta Gwl et Kin4 Pmapk pArpp PTyr.tif]

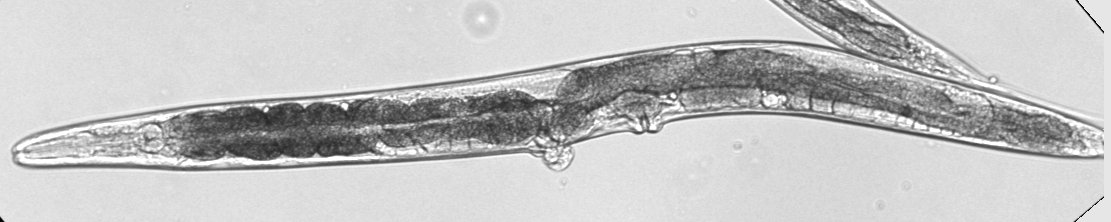

Supplement: Supplementary file 11 — Source data Fig. 8 [file 44318_2025_364_MOESM11_ESM.zip › Figure 8/8C/CTL/10x_PH_ctrl_1-2-1cut.jpg]

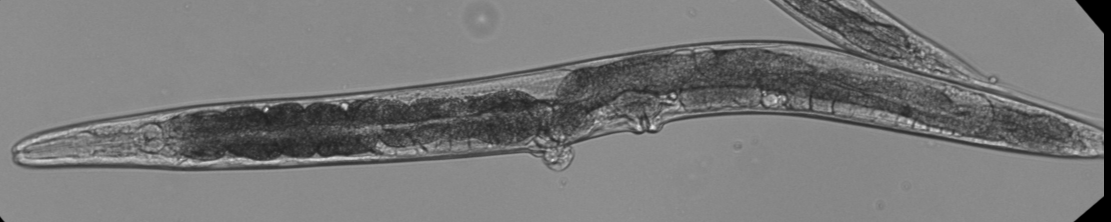

Supplement: Supplementary file 11 — Source data Fig. 8 [file 44318_2025_364_MOESM11_ESM.zip › Figure 8/8C/CTL/10x_PH_ctrl_1-2-1cut.tif]

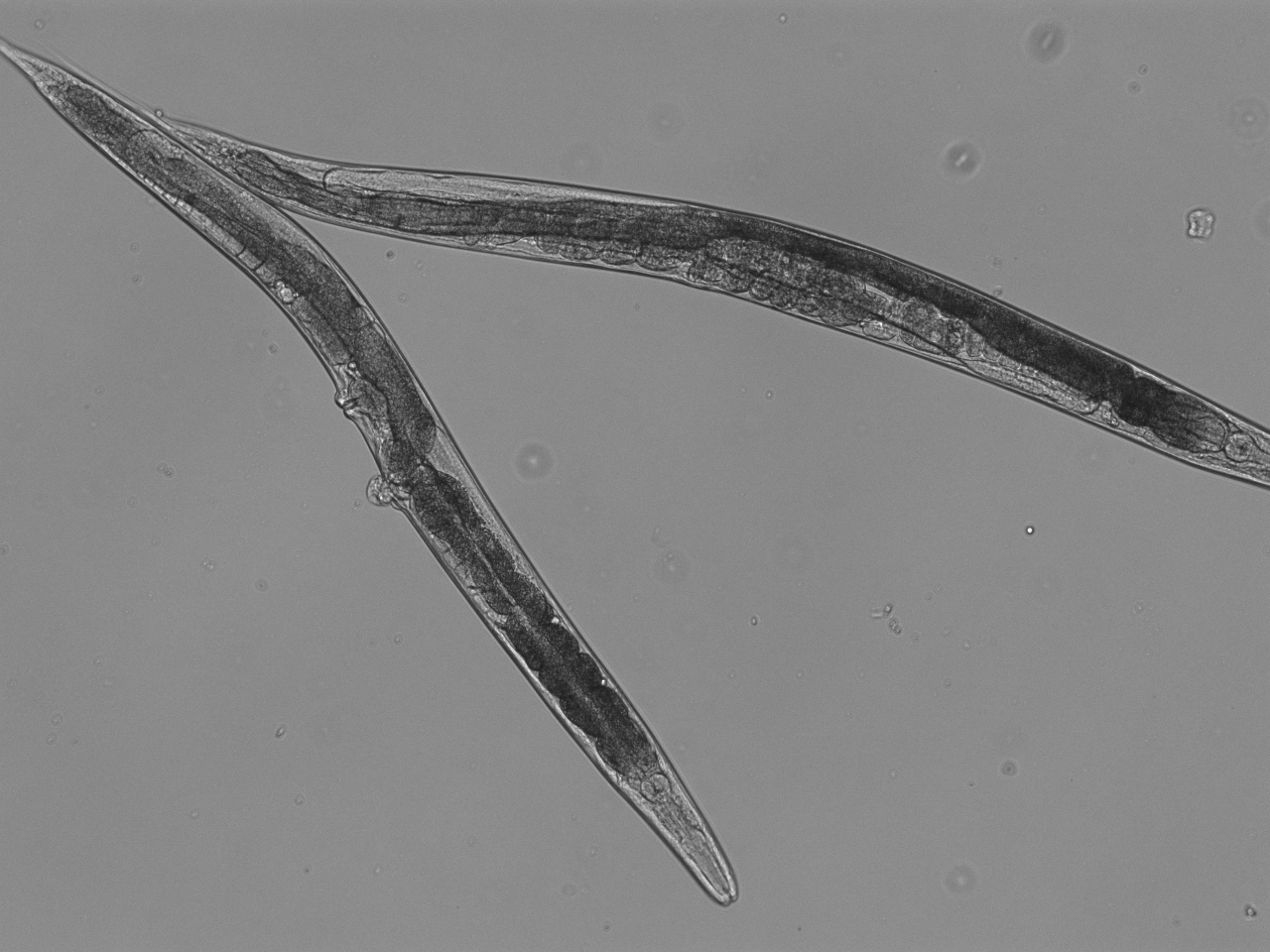

Supplement: Supplementary file 11 — Source data Fig. 8 [file 44318_2025_364_MOESM11_ESM.zip › Figure 8/8C/CTL/10x_PH_ctrl_1-2.tif]

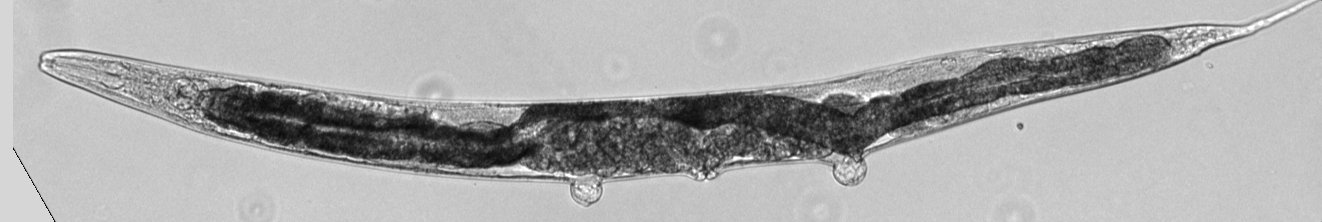

Supplement: Supplementary file 11 — Source data Fig. 8 [file 44318_2025_364_MOESM11_ESM.zip › Figure 8/8C/ensa-1/10x_PH_ensa-3-1cut.jpg]

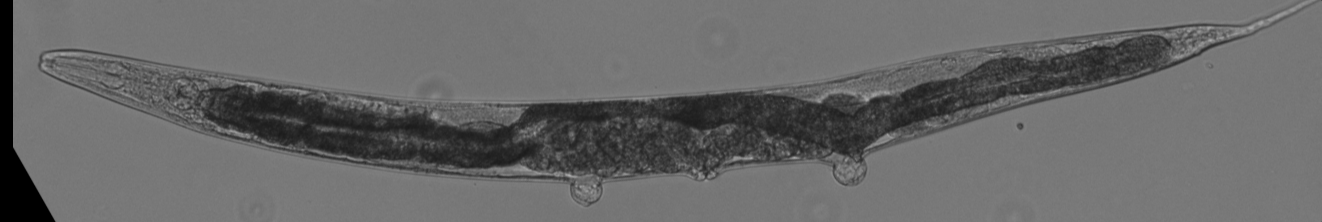

Supplement: Supplementary file 11 — Source data Fig. 8 [file 44318_2025_364_MOESM11_ESM.zip › Figure 8/8C/ensa-1/10x_PH_ensa-3-1cut.tif]

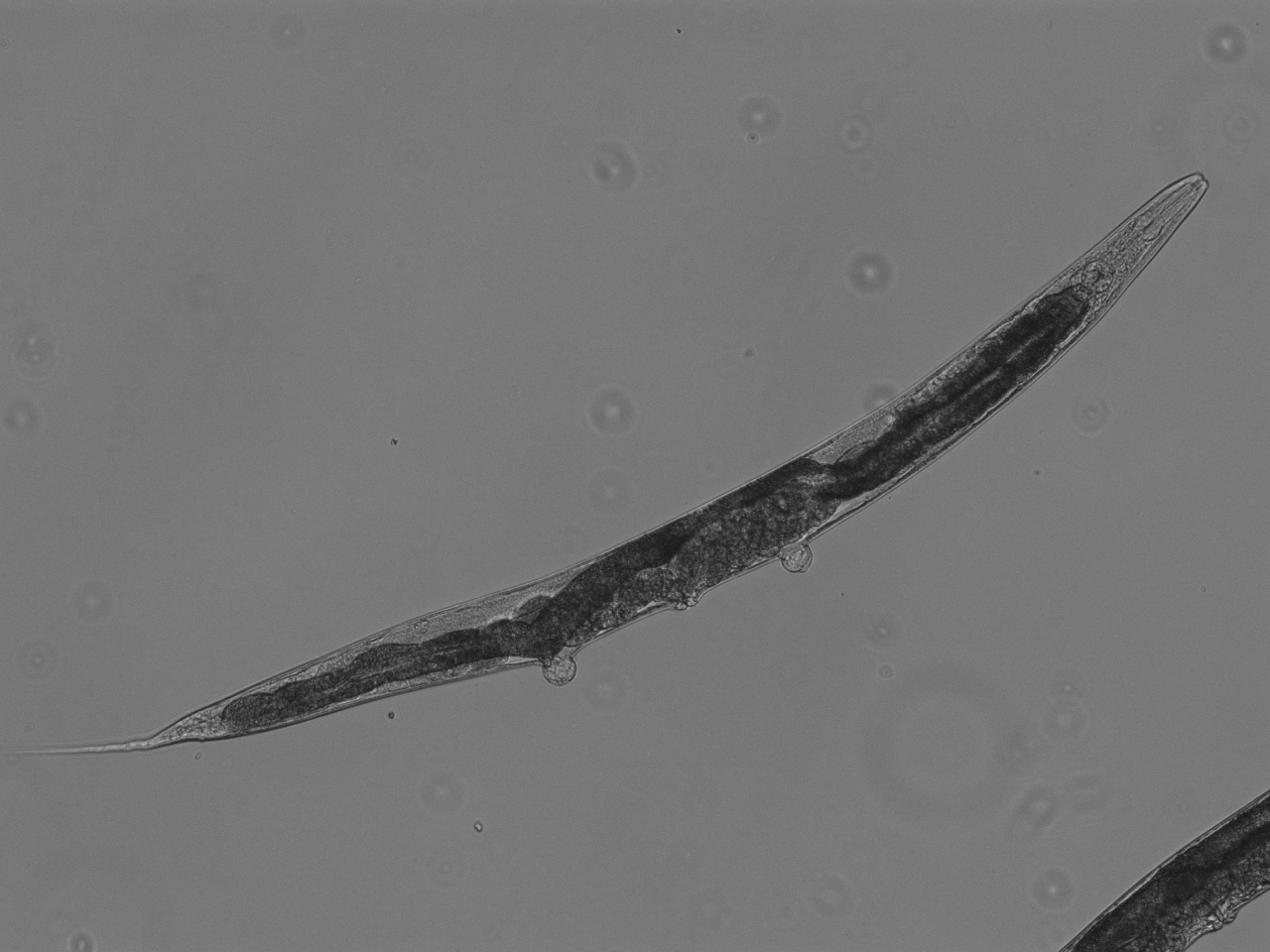

Supplement: Supplementary file 11 — Source data Fig. 8 [file 44318_2025_364_MOESM11_ESM.zip › Figure 8/8C/ensa-1/10x_PH_ensa-3.tif]

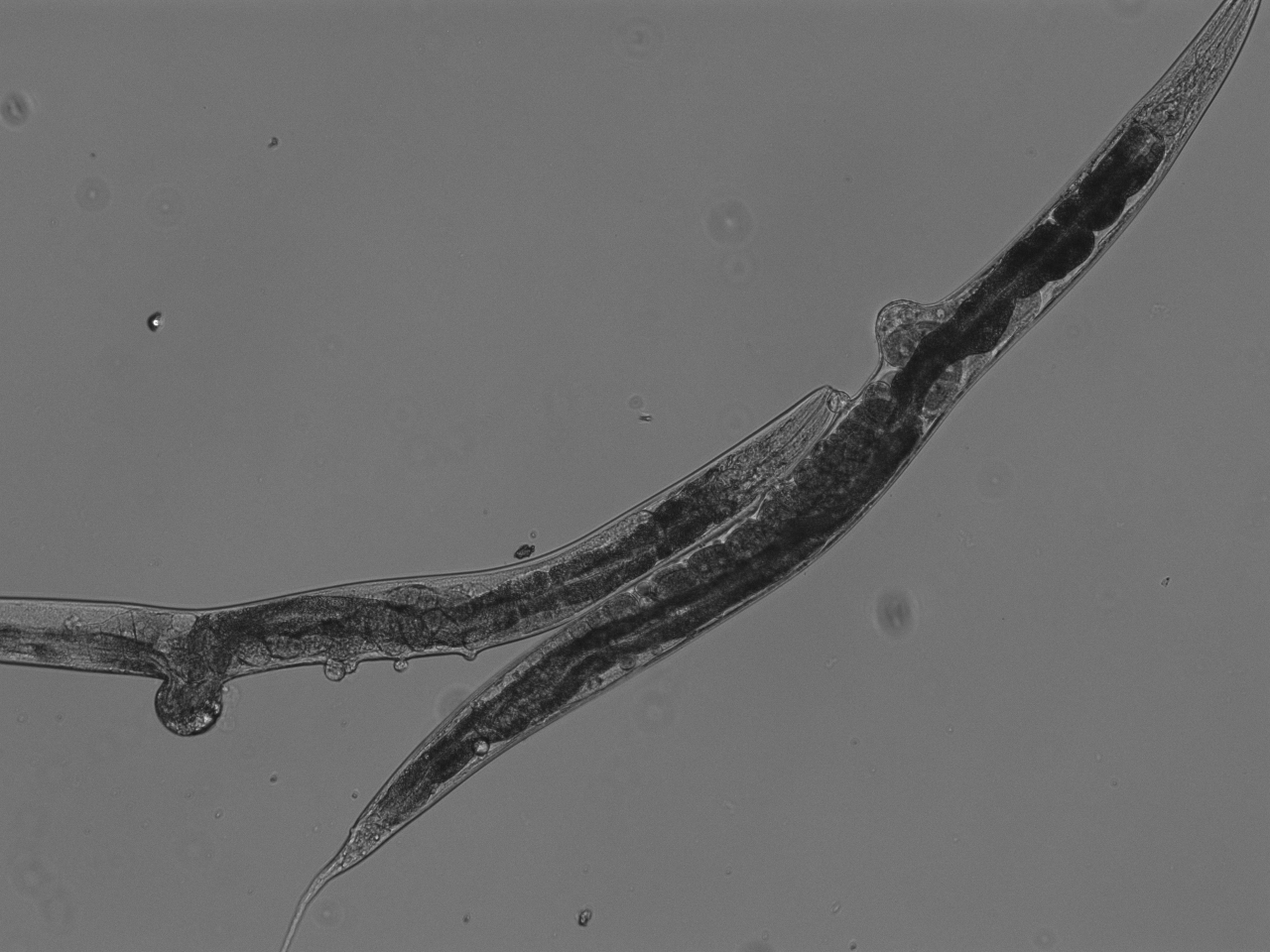

Supplement: Supplementary file 11 — Source data Fig. 8 [file 44318_2025_364_MOESM11_ESM.zip › Figure 8/8C/kin-4/10x_PH_kin4-1.tif]

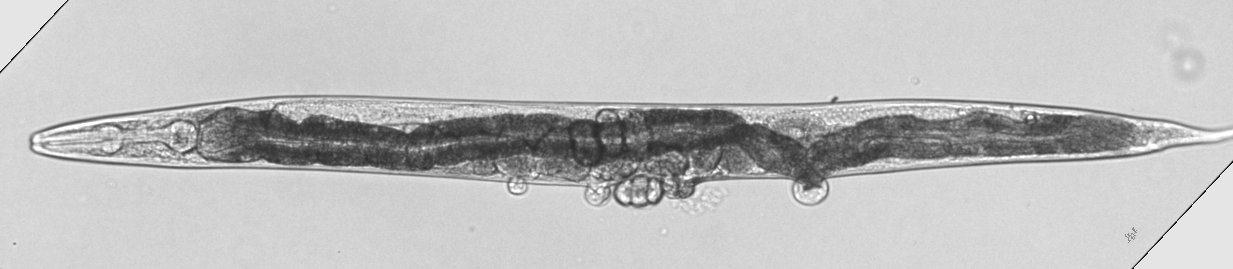

Supplement: Supplementary file 11 — Source data Fig. 8 [file 44318_2025_364_MOESM11_ESM.zip › Figure 8/8C/kin-4/10x_PH_kin4-4-1CUT.jpg]

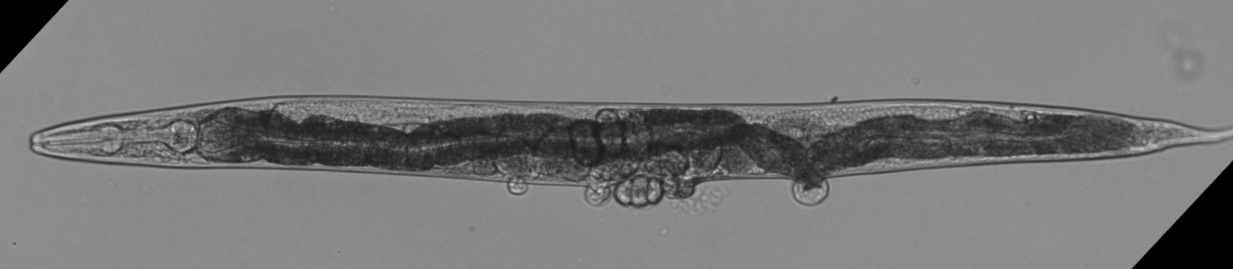

Supplement: Supplementary file 11 — Source data Fig. 8 [file 44318_2025_364_MOESM11_ESM.zip › Figure 8/8C/kin-4/10x_PH_kin4-4-1CUT.tif]

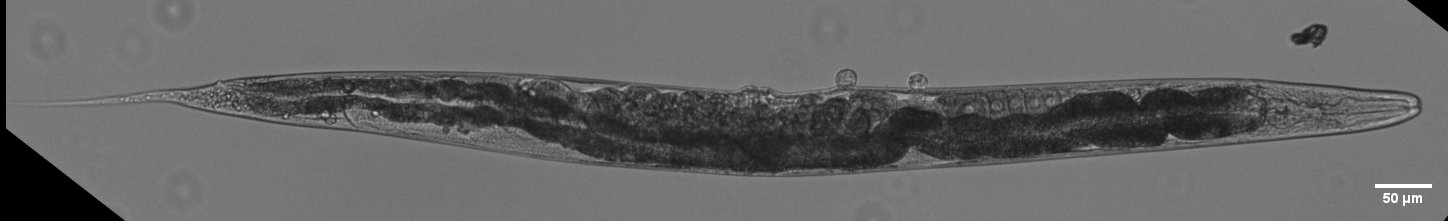

Supplement: Supplementary file 11 — Source data Fig. 8 [file 44318_2025_364_MOESM11_ESM.zip › Figure 8/8C/scale pic.jpg]

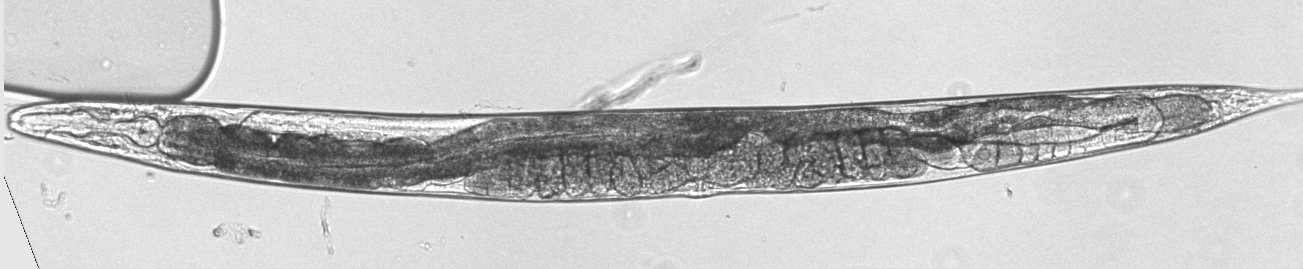

Supplement: Supplementary file 11 — Source data Fig. 8 [file 44318_2025_364_MOESM11_ESM.zip › Figure 8/8C/sur-6/10x_PH_sur6_3-1CUT.jpg]

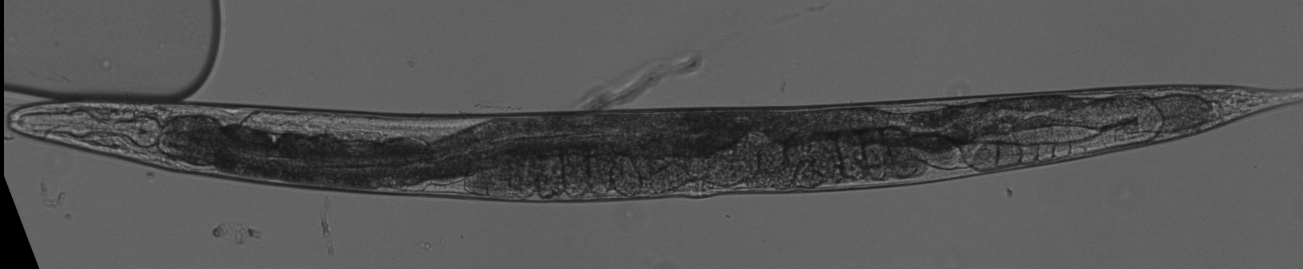

Supplement: Supplementary file 11 — Source data Fig. 8 [file 44318_2025_364_MOESM11_ESM.zip › Figure 8/8C/sur-6/10x_PH_sur6_3-1CUT.tif]

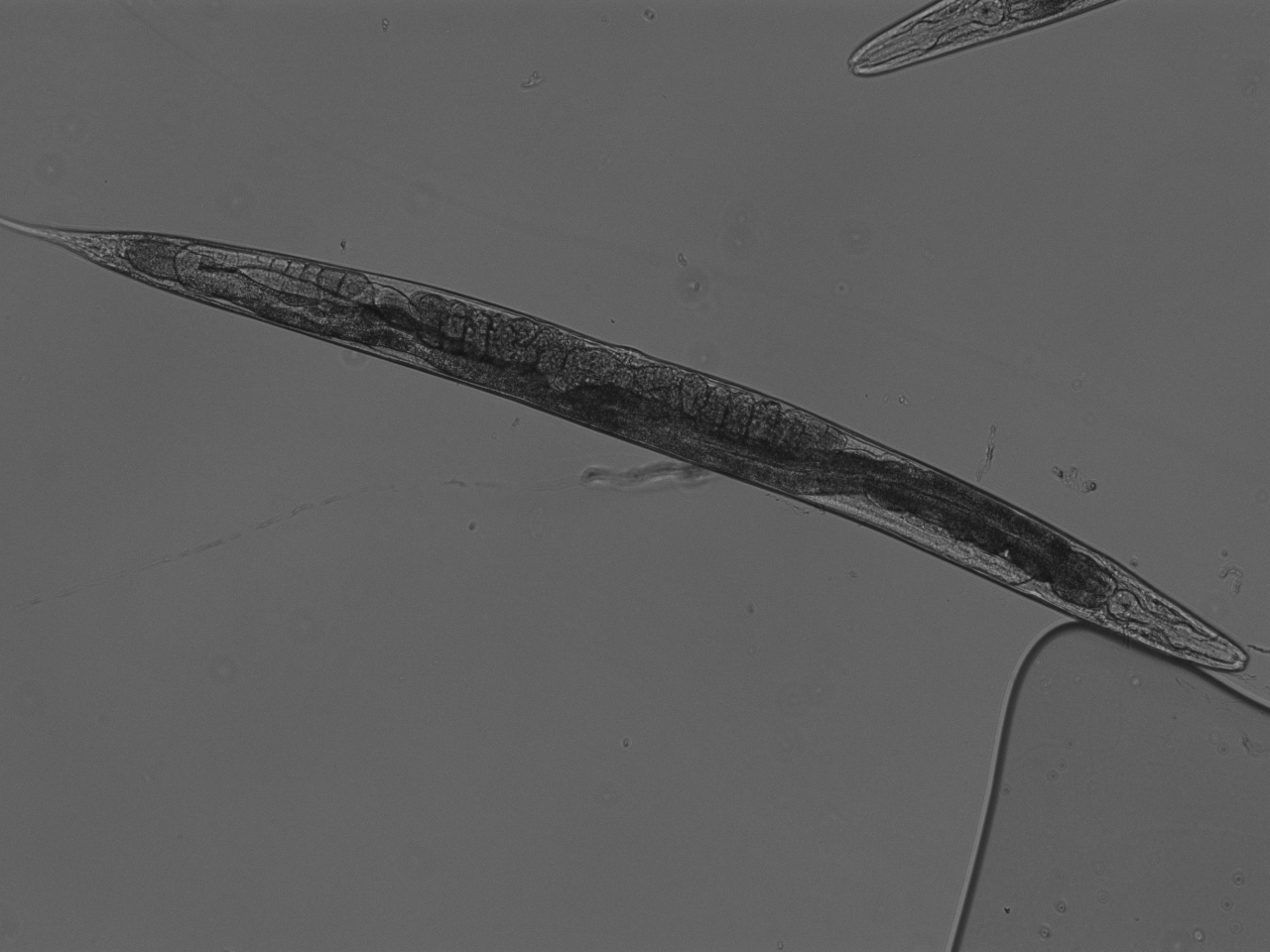

Supplement: Supplementary file 11 — Source data Fig. 8 [file 44318_2025_364_MOESM11_ESM.zip › Figure 8/8C/sur-6/10x_PH_sur6_3.tif]

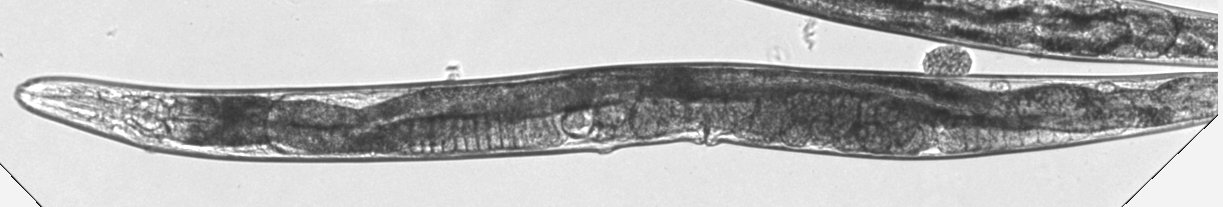

Supplement: Supplementary file 11 — Source data Fig. 8 [file 44318_2025_364_MOESM11_ESM.zip › Figure 8/8C/sur-6 kin-4/sur-6_kin-4_10PH_1-1.jpg]

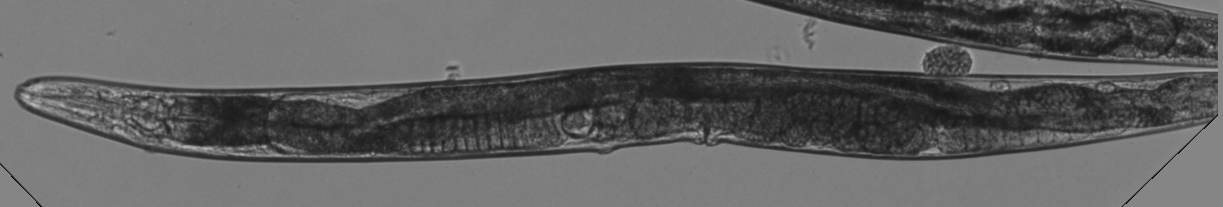

Supplement: Supplementary file 11 — Source data Fig. 8 [file 44318_2025_364_MOESM11_ESM.zip › Figure 8/8C/sur-6 kin-4/sur-6_kin-4_10PH_1-1.tif]

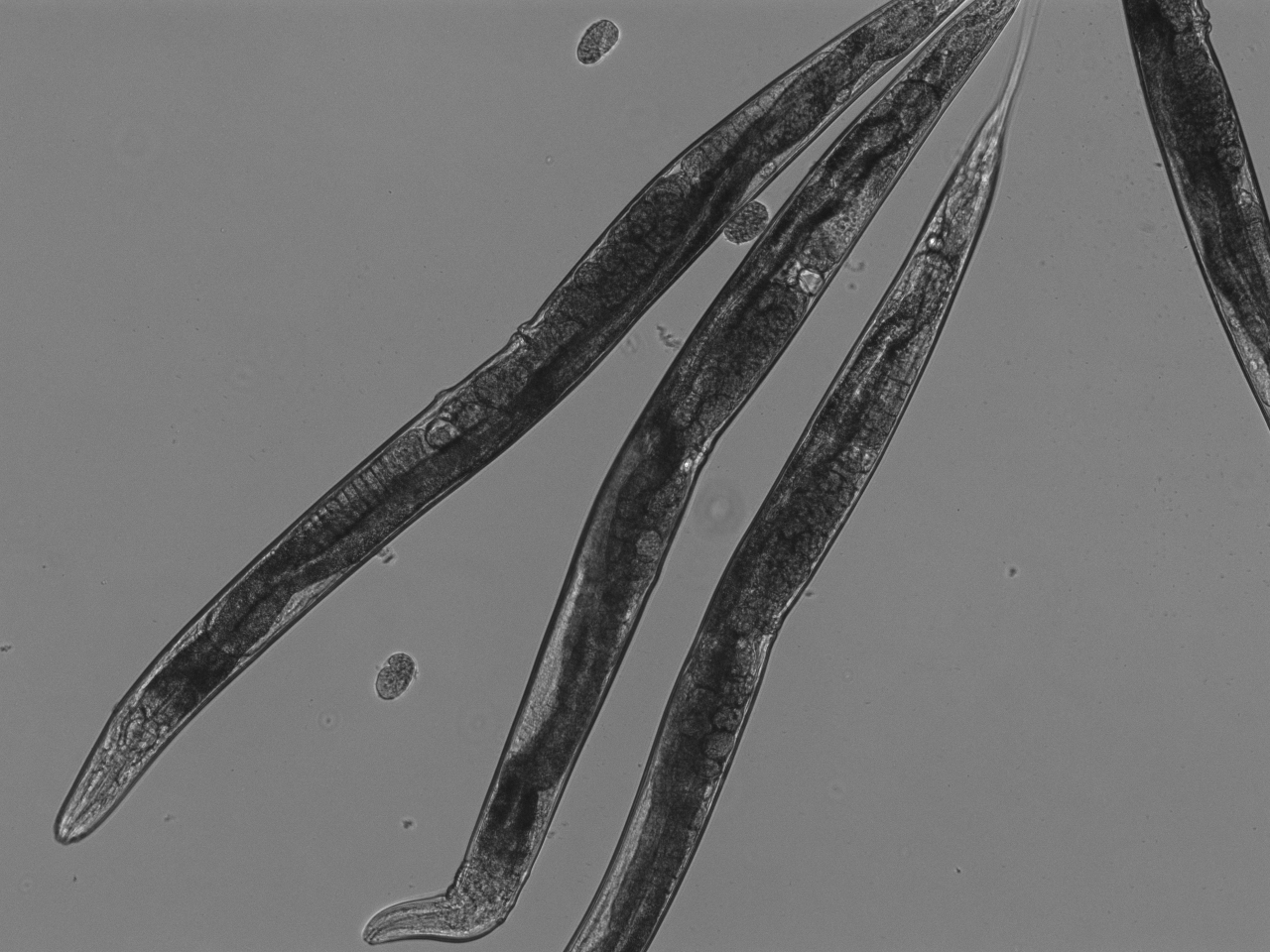

Supplement: Supplementary file 11 — Source data Fig. 8 [file 44318_2025_364_MOESM11_ESM.zip › Figure 8/8C/sur-6 kin-4/sur-6_kin-4_10PH_1.tif]

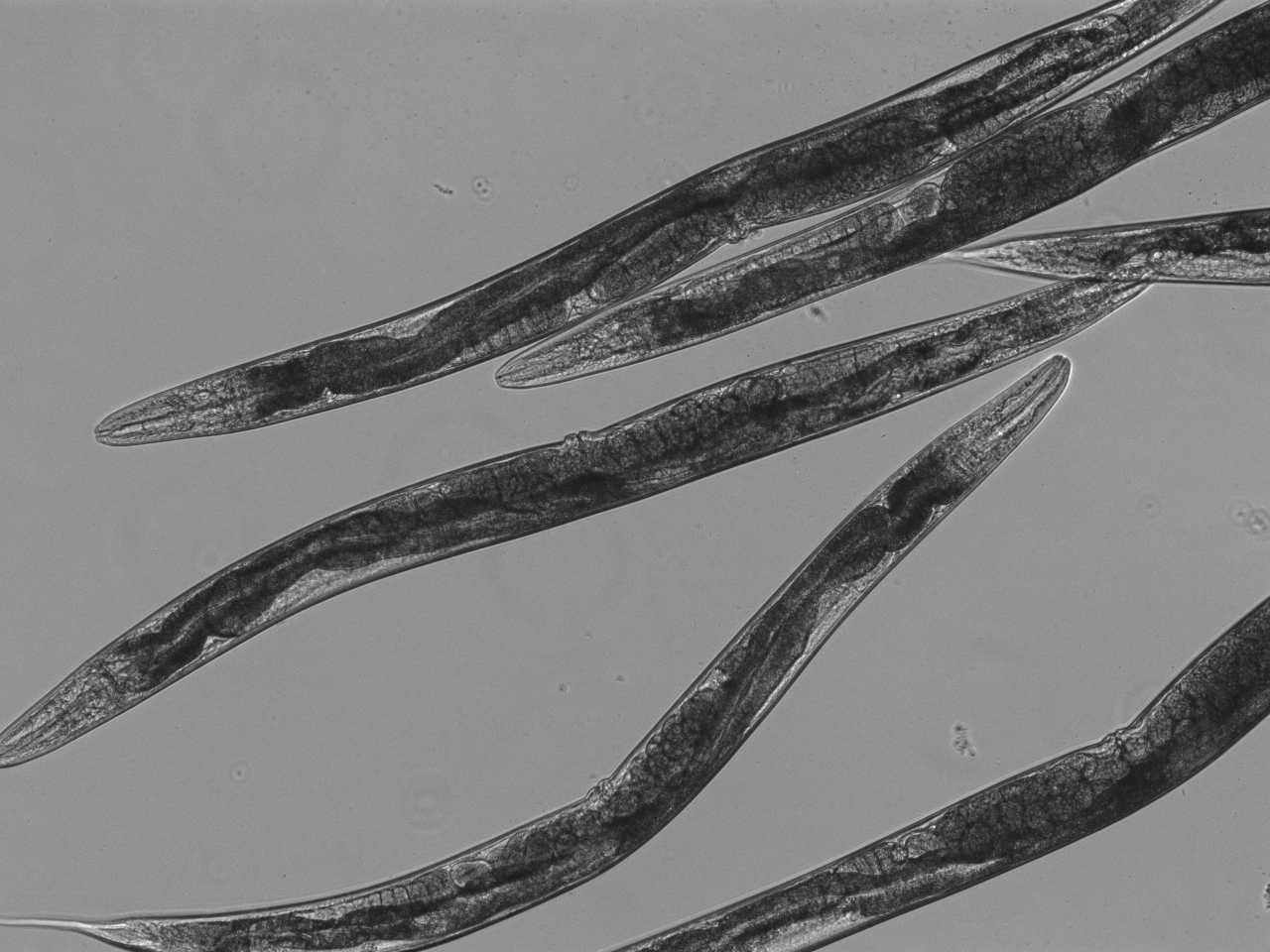

Supplement: Supplementary file 11 — Source data Fig. 8 [file 44318_2025_364_MOESM11_ESM.zip › Figure 8/8C/sur6 ensa-1/sur-6_ensa-1_10PH_1.tif]

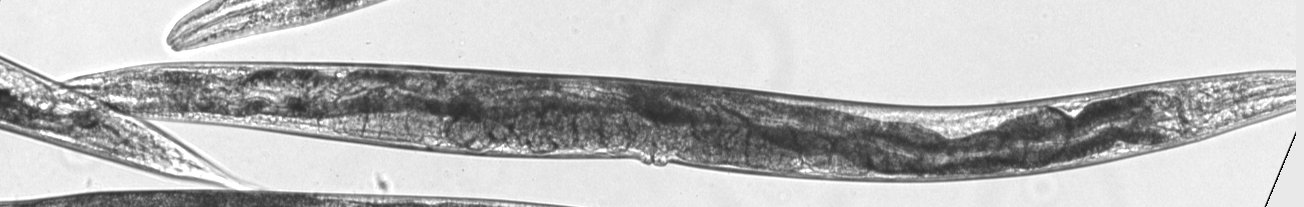

Supplement: Supplementary file 11 — Source data Fig. 8 [file 44318_2025_364_MOESM11_ESM.zip › Figure 8/8C/sur6 ensa-1/sur-6_ensa-1_10PH_1crop-1.jpg]

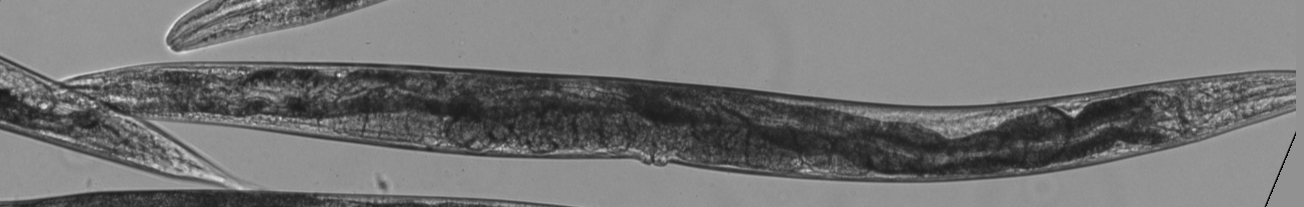

Supplement: Supplementary file 11 — Source data Fig. 8 [file 44318_2025_364_MOESM11_ESM.zip › Figure 8/8C/sur6 ensa-1/sur-6_ensa-1_10PH_1crop-1.tif]
