# Supplementary material for: The MAST kinase KIN-4 carries out mitotic entry functions of Greatwall in C. elegans
Source: EMBO J. 2025 Feb 17;44(7):1943–74. doi: 10.1038/s44318-025-00364-w (PMC11961639; doi:10.1038/s44318-025-00364-w)
Supplement: Supplementary file 12 — Expanded View Figures [file 44318_2025_364_MOESM12_ESM.pdf]

Expanded View Figures

Figure EV1. *sur-6* inactivation affects the duration of mitosis in the AB blastomere.

▶

(**A**) Schematic of the first and second cell divisions of *C. elegans* embryos. The arrows show the ingression of the cytokinetic furrows in P_0, AB, and P_1 blastomeres. The dashed circle line shows nuclear envelope permeabilization. (**B**) Graphs presenting the duration of interphase (time between furrow ingression in P_0 to nuclear envelope permeabilization in AB and P_1 blastomeres) and mitosis (time between nuclear envelope permeabilization in AB and P1 to furrow ingression) in wild-type and *sur-6*(sv30) hetero (-/+) and homozygous mutants (-/-). *n* number of embryos analyzed. Non-parametric tests (Kruskal–Wallis) were used to calculate *p* values, which are displayed as follows: ns = $p > 0.05$; * = $p < 0.05$; **** = $p < 0.0001$. Exact *p* values from Interphase AB (L-R); $p < 0.0001$, $p < 0.0001$; Interphase P_1 (L-R); $p < 0.0001$, $p < 0.0001$, Mitosis AB (L-R) ; $p < 0.0001$, $p = 0.0116$, Mitosis P_1 (L-R); ns. Error bars display the standard error to the mean. ns no-significant differences. (**C**) Graphs presenting the duration of interphase (time between furrow ingression in P_0 to nuclear envelope permeabilization in AB and P_1 blastomeres) and mitosis (time between nuclear envelope permeabilization in AB and P1 to furrow ingression) in wild-type and *sur-6ts* mutants. n=number of embryos analyzed. Unpaired *T*-test was used to calculate *p* values, which are displayed as follows: ns = $p > 0.05$; *** = $p < 0.001$; **** = $p < 0.0001$. Exact *p* values from Interphase AB; $p < 0.0001$, Interphase P_1; $p < 0.0001$, Mitosis AB; $p < 0.0004$, Mitosis P_1 (L-R); ns. Error bars display the standard error to the mean. ns no-significant differences.

© The Author(s)

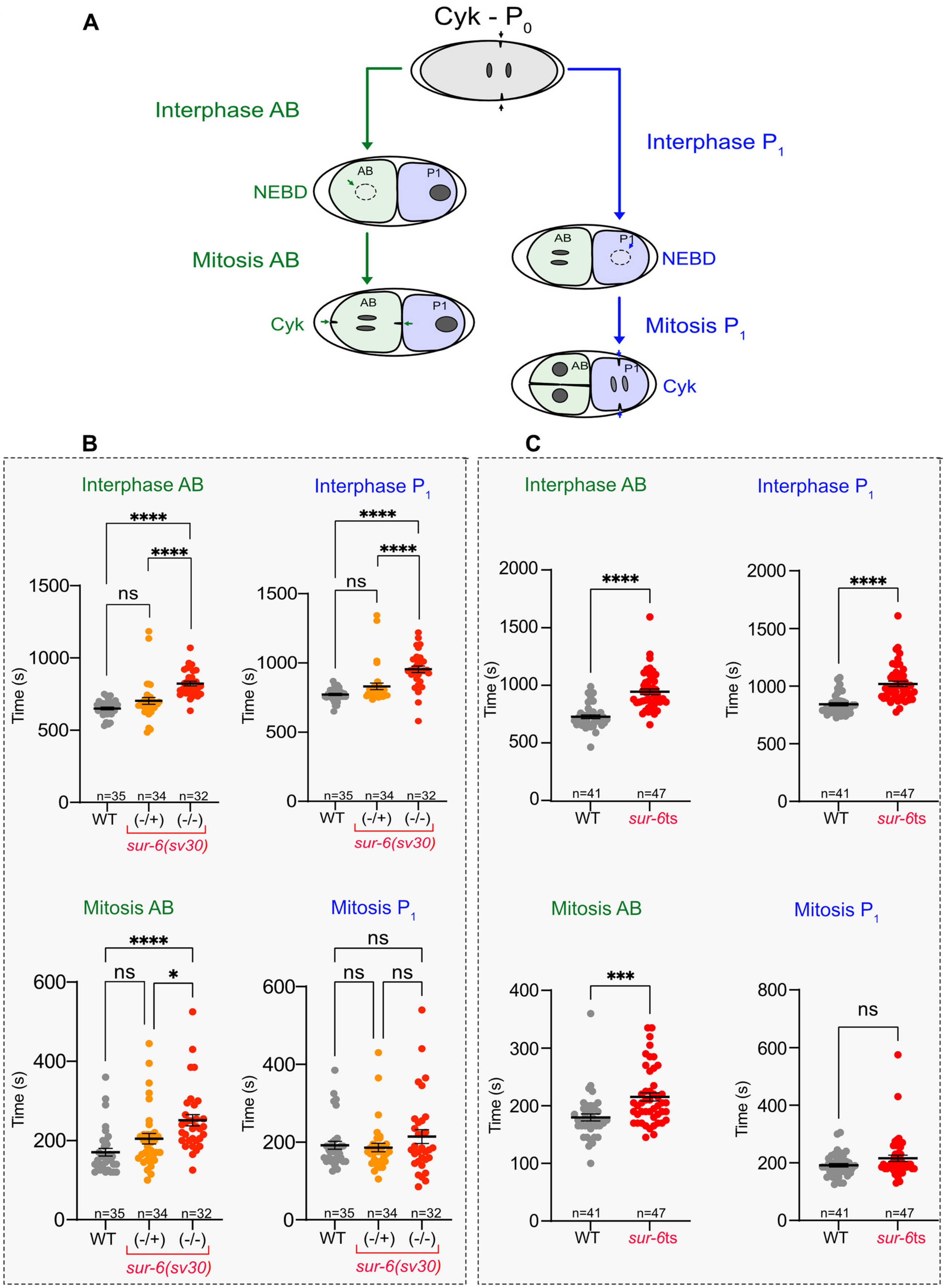
A
Cyk - P0
Interphase AB
NEBD
AB
P1
Mitosis AB
Cyk
Interphase P1
NEBD
Mitosis P1
Cyk
B
Interphase AB
Interphase P1
Mitosis AB
Mitosis P1
Time (s)
n=35 n=34 n=32
WT (-/+) (-/-)
sur-6(sv30)
****
ns
*
C
n=41 n=47
WT sur-6ts
***

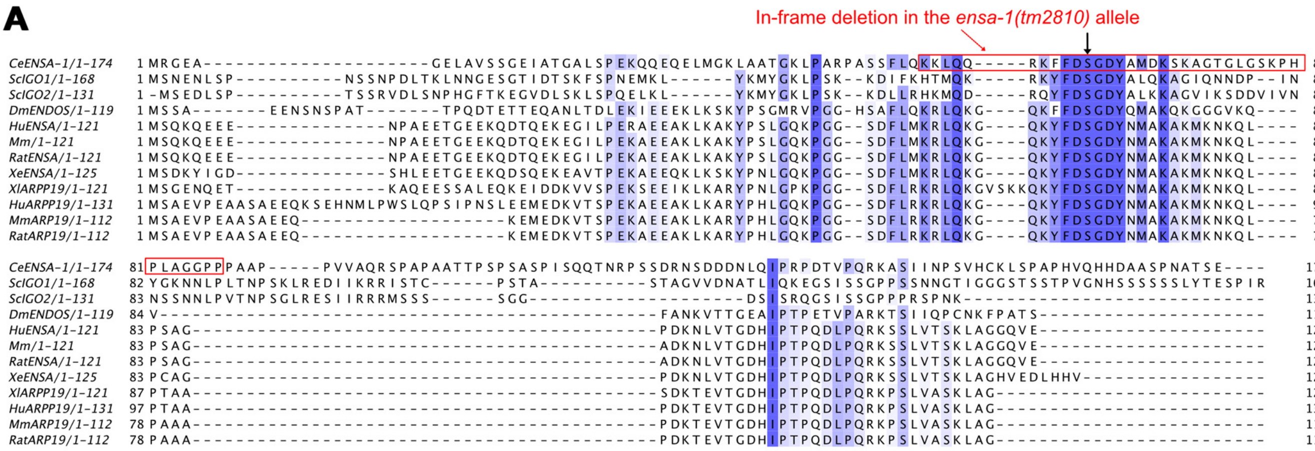

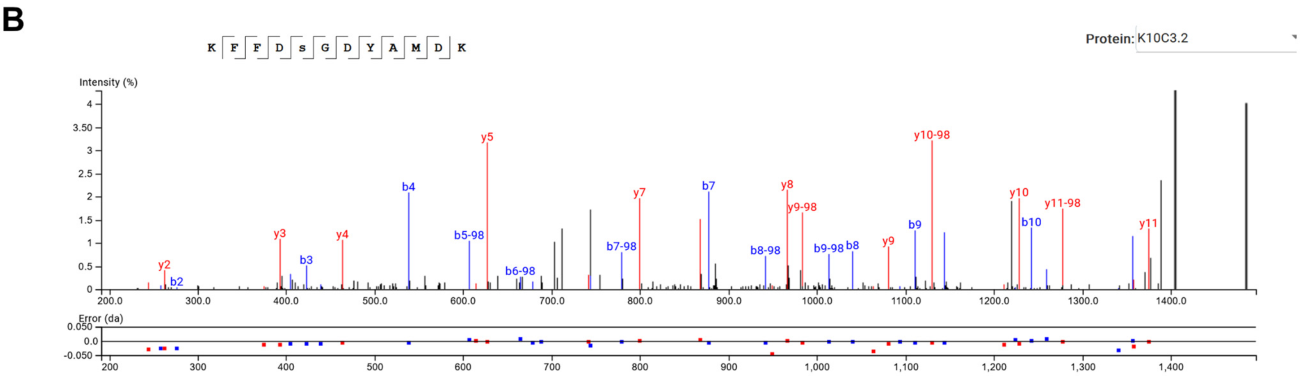

#	b	b-H2O	b-NH3	b-Neutral Loss	b(2+)	Seq	y	y-H2O	y-NH3	y-Neutral Loss	y(2+)	#
1	129.10	111.09	112.08		65.05	K						12
2	276.19	258.18	259.14		138.59	F	1375.49	1357.48	1358.48	1277.51	688.24	11
3	423.24	405.23	406.21		212.12	F	1228.43	1210.41	1211.40	1130.45	614.71	10
4	538.27	520.26	521.24		269.63	D	1081.36	1063.37	1064.33	983.38	541.18	9
5	705.26	687.25	688.24	607.28	353.13	S(+79.97)	966.32	948.32	949.34	868.34	483.66	8
6	762.29	744.29	745.26	664.30	381.64	G	799.32	781.32	782.30		400.16	7
7	877.31	859.30	860.29	779.33	439.16	D	742.30	724.30	725.28		371.65	6
8	1040.37	1022.37	1023.35	942.40	520.69	Y	627.28	609.27	610.25		314.14	5
9	1111.42	1093.40	1094.39	1013.43	556.21	A	464.22	446.21	447.19		232.61	4
10	1242.45	1224.43	1225.43	1144.48	621.73	M	393.19	375.17	376.15		197.09	3
11	1357.48	1339.47	1340.48	1259.49	679.24	D	262.16	244.15	245.11		131.57	2
12						K	147.11	129.10	130.09		74.06	1

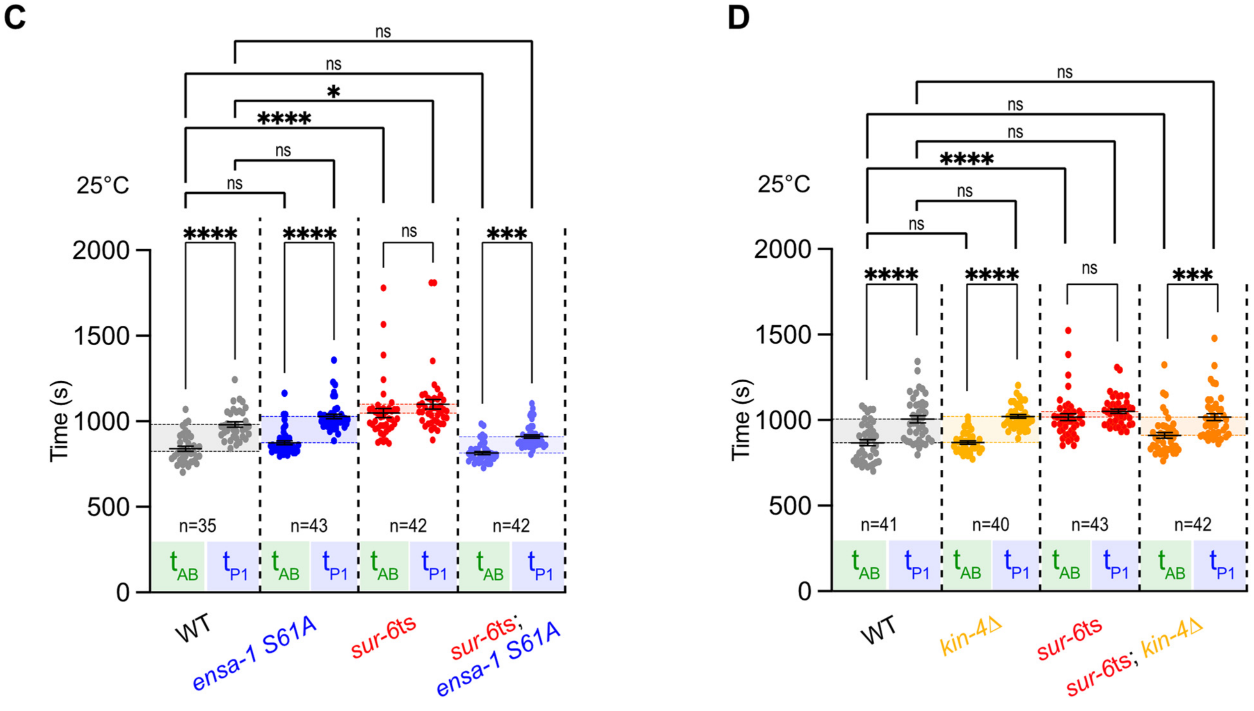

 © The Author(s)

Figure EV2. KIN-4-dependent ENSA-1 phosphorylation at the DSG sequence motif regulates SUR-6$^{PP2A\text{-}B55}$ activity.

(**A**) Multiple protein sequence alignments of Endosulfine and Arpp19 from several species (H.s. *Homo sapiens*, X. l, *Xenopus laevis*, M. m. *Mus musculus*, R. n., *Rattus Norvegicus*, D. m. *Drosophila melanogaster*, C. e. *Caenorhabditis elegans, S. c. Saccharomyces cerevisiae*). Endosulfine and Arpp19-related proteins in *S. cerevisiae* are termed Igo1 and Igo2. Note that the DSG sequence motif and surrounding residues are highly evolutionarily conserved. The *ensa-1(tm2810)* allele encodes a truncated protein with an in-frame deletion, removing the DSG sequence motif and surrounding residues. (**B**) Representative MS/MS spectrum confirming ENSA-1 phosphorylation at site S61. The peptide sequence containing S61 indicates singly charged fragment ions (y + -ion and b + -ion series). The table at the bottom shows the theoretical mass for each fragment ion and the experimentally detected b+ (blue) and y + -ions (red). (**C**, **D**) Graph presenting the cell cycle length of AB (light green) and P_1 blastomeres (light blue) in embryos of the indicated genotype represented as the mean ± standard error to the mean. *n* number of embryos analyzed. Non-parametric tests (Kruskal−Wallis) were used to calculate *p* values, which are displayed as follows: ns = $p > 0.05$; * = $p < 0.05$; ** = $p < 0.01$; *** = $p < 0.001$; **** = $p < 0.0001$. Exact *p* values from (**C**) (L-R); $p < 0.0001$, $p < 0.0001$, $p < 0.0001$, $p = 0.0280$, $p = 0.0003$. Exact *p* values from (**D**) (L-R); $p < 0.0001$, $p < 0.0001$, $p < 0.0001$, $p = 0.0004$. Error bars display the standard error to the mean. ns no-significant differences.

© The Author(s)

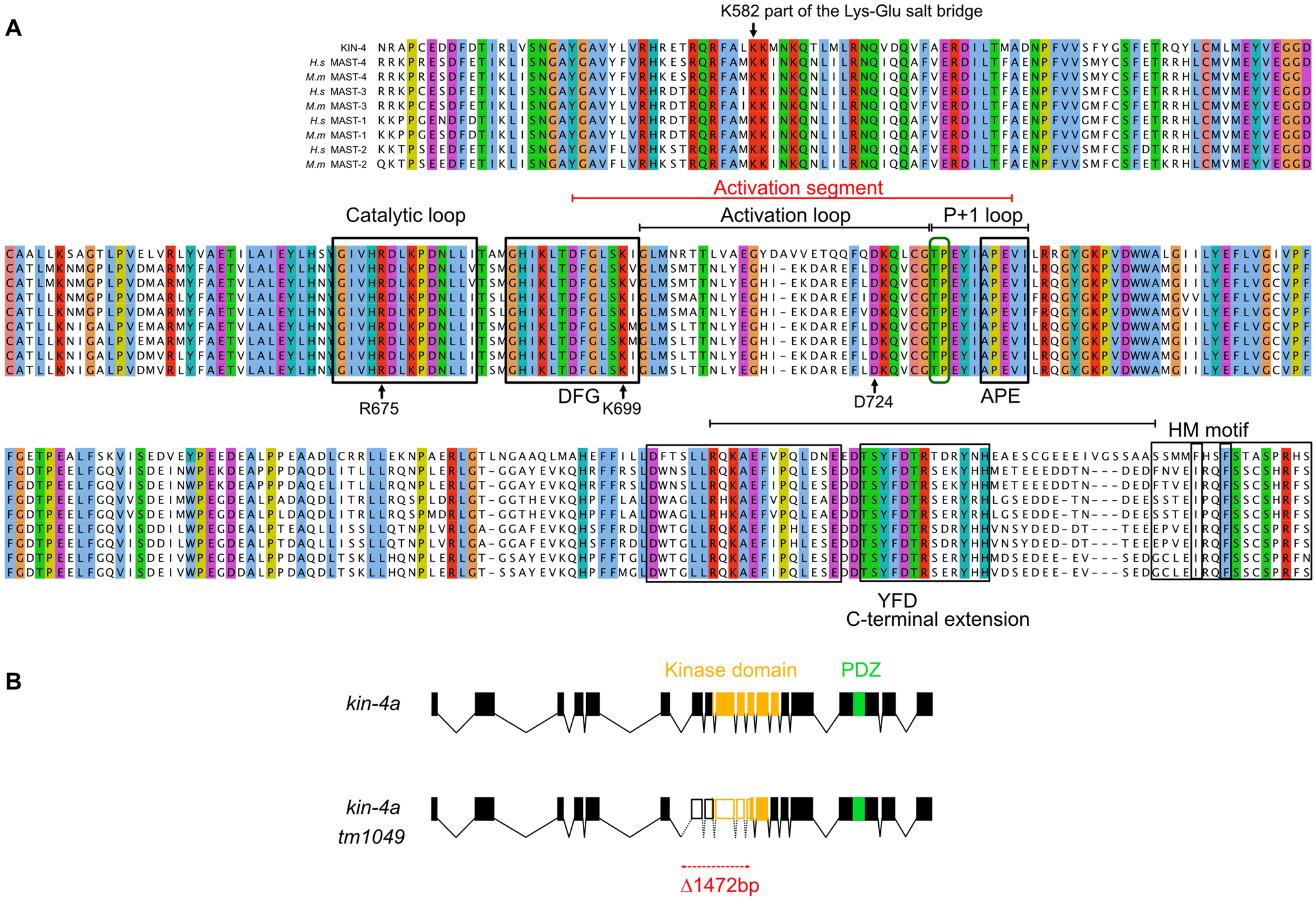

Figure EV3. Multiple protein sequence alignments of the MAST kinase domain.

(**A**) Protein sequences corresponding to the kinase domains of MAST 1, 2, 3, 4 kinases from *Homo sapiens (H. s), Mus musculus (M. m)* were aligned with the kinase domain of KIN-4 from *Caenorhabditis elegans (C. e)*. Sequences were aligned using Clustal Omega and visualized with Jalview. The location of the catalytic-, Mg-binding-, P + 1-, activation loops, and C-terminal extension with the hydrophobic motif (HM) are indicated. (**B**) *kin-4a* gene structure. The *kin-4(tm1049)* allele deletes 1472 bp in the kinase domain (orange).

 © The Author(s)

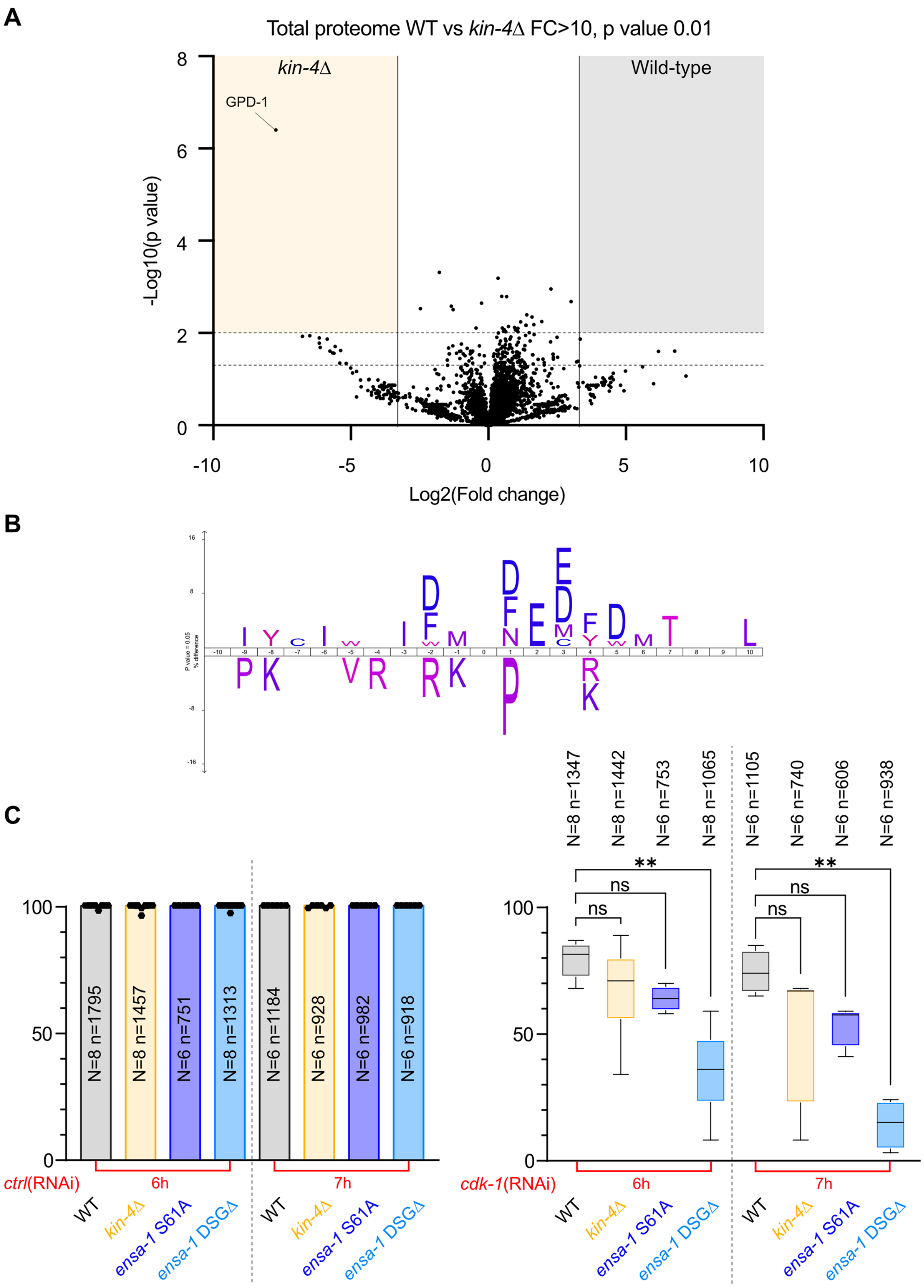
A
Total proteome WT vs kin-4Δ FC>10, p value 0.01
kin-4Δ
Wild-type
GPD-1
-Log10(p value)
Log2(Fold change)
B
C
N=8 n=1795
N=8 n=1457
N=6 n=751
N=8 n=1313
N=6 n=1184
N=6 n=928
N=6 n=982
N=6 n=918
ctrl(RNAi)
6h
7h
WT
kin-4Δ
ensa-1 S61A
ensa-1 DSGΔ
N=8 n=1347
N=8 n=1442
N=6 n=753
N=8 n=1065
N=6 n=1105
N=6 n=740
N=6 n=606
N=6 n=938
ns
**
cdk-1(RNAi)

Figure EV4. Quantitative mass spectrometry analysis of wild-type N2 versus *kin-4*Δ proteome.

(**A**) Visualization of the quantitative proteomic analysis of wild-type N2 versus *kin-4Δ* in a Volcano plot. Each point on the graph represents a peptide. The $\log_2$-fold change differences between the WT and *kin-4Δ* were plotted on the x-axis, and the −log10 *p* value differences were plotted on the y-axis. Peptides whose abundance is increased in wild-type versus *kin-4Δ* are located to the right of zero on the x-axis, while peptides whose abundance is decreased are illustrated to the right of zero. Peptides with statistically significant differential abundance lie above the horizontal threshold ($p = 0.01$). The horizontal dashed lines represent a *p* value of 0.01 and 0.05 (Student's bilateral *t*-test and assuming equal variance between groups, see also methods section), and the vertical dashed lines show a fold change between WT and *kin-4Δ* of 10. (**B**) IceLogo representation of the phosphopeptide sequences over-represented in *kin-4*Δ strain compared to the total phosphorylated sequences identified in the analysis (Fig. 5B). Significantly over- and under-represented amino acids are visualized. The position 0 corresponds to the position of the phosphorylated serine or threonine. (**C**) Graph and Box plot showing the percentage of embryonic viability of wild-type N2, *kin-4Δ*, *ensa-1 S61A*, and *ensa-1Δ* exposed to control (Ctrl) or *cdk-1*(RNAi) for 6 or 7 h. *N* is the number of independent experiments, and *n* is the total number of embryos counted. A non-parametric test (Kruskal−Wallis) was used to calculate *p* values displayed as follows: ns = $p > 0.05$; ** = $p < 0.01$, n.s not significant. Exact *p* values from (L-R) $p = 0.0020$; $p = 0.092$. The box plot indicates the median and interquartile ranges (25th−75th percentile) with whiskers representing min to max values.

 © The Author(s)

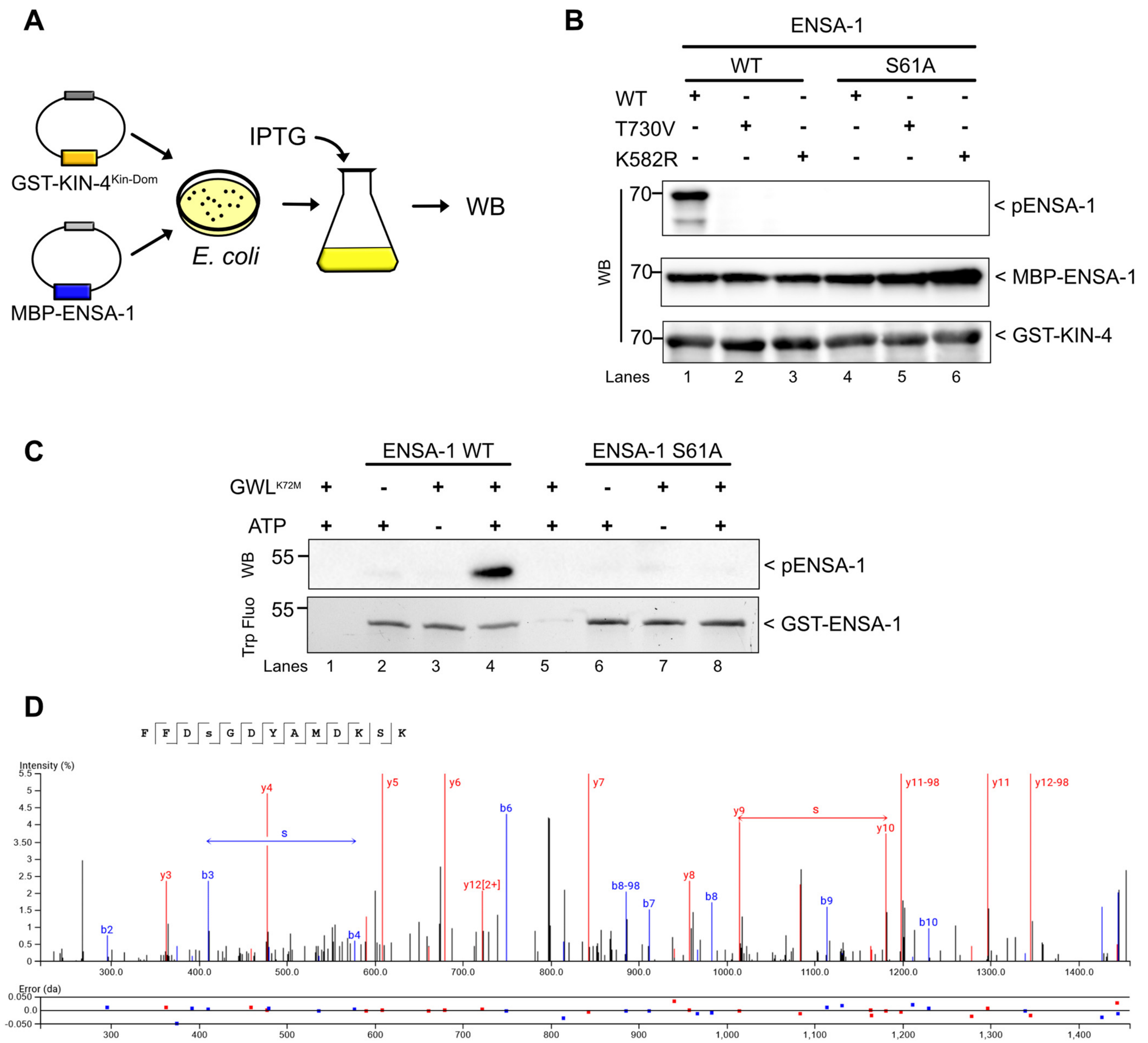

#	b	b-H2O	b-NH3	b-Neutral Loss	b(2+)	Seq	y	y-H2O	y-NH3	y-Neutral Loss	y(2+)	#
1	148.08	130.07	131.05		74.54	F						13
2	295.13	277.13	278.12		148.07	F	1443.52	1425.54	1426.52	1345.58	722.27	12
3	410.16	392.15	393.14		205.59	D	1296.47	1278.49	1279.45	1198.50	648.74	11
4	577.16	559.16	560.14	479.18	289.08	S(+79.97)	1181.45	1163.44	1164.44	1083.48	591.23	10
5	634.19	616.18	617.16	536.21	317.60	G	1014.45	996.45	997.43		507.73	9
6	749.21	731.21	732.19	651.24	375.15	D	957.43	939.42	940.37		479.18	8
7	912.28	894.27	895.25	814.33	456.64	Y	842.41	824.40	825.38		421.70	7
8	983.32	965.31	966.30	885.34	492.16	A	679.34	661.33	662.32		340.17	6
9	1114.34	1096.35	1097.33	1016.38	557.68	M	608.30	590.29	591.28		304.65	5
10	1229.37	1211.35	1212.36	1131.39	615.19	D	477.26	459.24	460.24		239.13	4
11	1357.48	1339.47	1340.45	1259.50	679.24	K	362.22	344.23	345.21		181.62	3
12	1444.52	1426.52	1427.49	1346.54	722.76	S	234.14	216.13	217.12		117.57	2
13						K	147.11	129.10	130.09		74.06	1

Figure EV5. KIN-4 phosphorylates ENSA-1 at the DSG motif in vitro.

(**A**) Schematic of the approach to test ENSA-1 phosphorylation by KIN-4$^{Kin\text{-}dom}$ directly in *E. coli*. Plasmids with different replication origins expressing GST-KIN-4$^{Kin\text{-}dom}$ and GST-ENSA-1 wild-type or variants were co-expressed in the *E. coli* BL21 strain (left panel). After protein induction with IPTG, total bacterial lysates were prepared in Laemmli sample buffer, and proteins were separated by SDS-PAGE before transfer on nitrocellulose membrane for western blot analysis using antibodies directed against pENSA-1, MBP, and GST (from top to bottom, right panel). (**B**) Western blot analysis of kinase reactions was carried out with Xenopus Gwl^{K72M} and GST-ENSA-1 WT or S61A as substrate. Blots were probed with antibodies to the phospho-DSG motif. The lower panel shows GST-ENSA-1 protein levels detected by tryptophane fluorescence (stain-free, Bio-Rad). (**C**) Representative MS/MS spectrum confirming ENSA-1 phosphorylation at site S61 site after in vitro phosphorylation by KIN-4. The peptide sequence containing S61 indicates singly charged fragment ions (y + -ion and b + -ion series). (**D**) The table shows the theoretical mass for each fragment ion and the experimentally detected b+ (blue) and y + -ions (red).

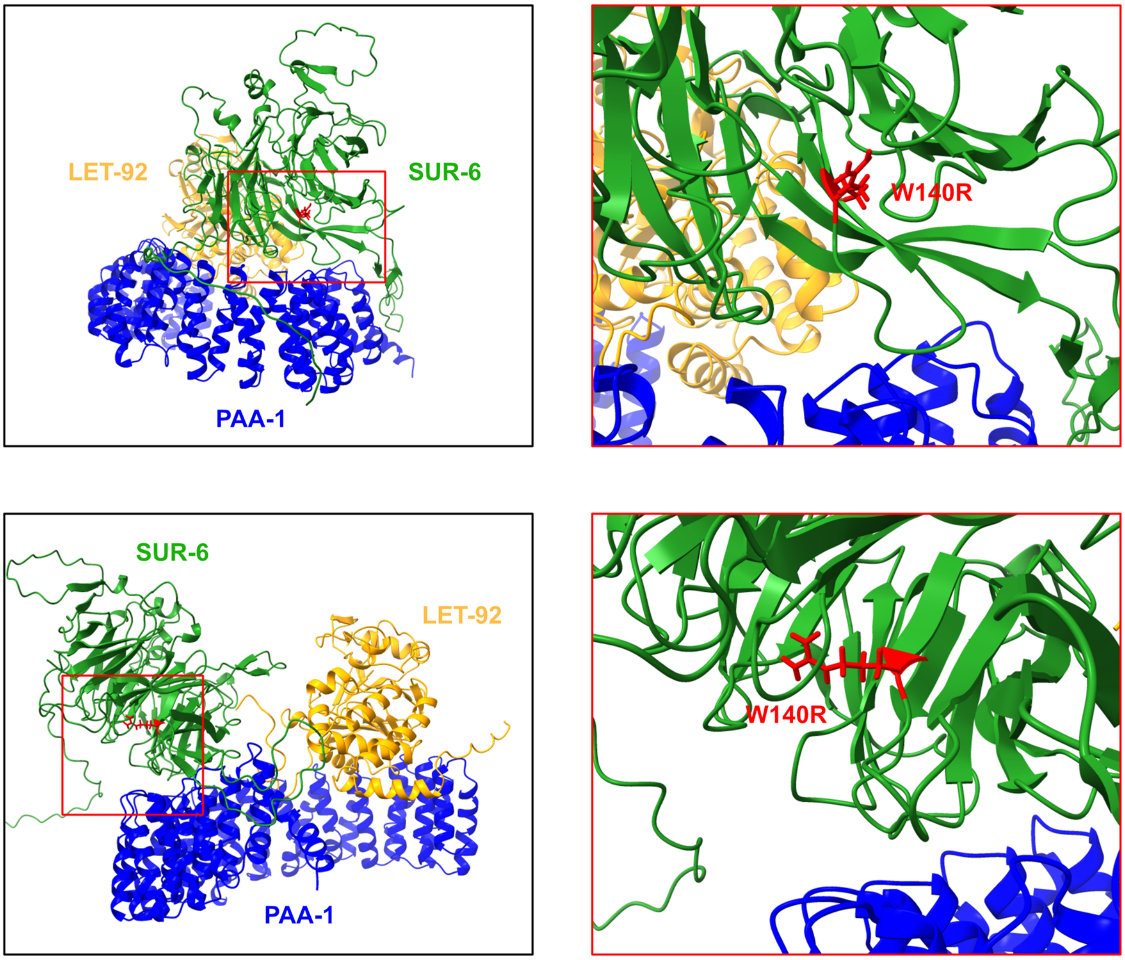

Figure EV6. Alphafold model of *C. elegans* PP2A-B55^{SUR-6} complex harboring the SUR-6 W140R mutation.

The worm PP2A-B55 phosphatase complex contains the B55 subunit SUR-6 (green), the scaffold PAA-1 (blue), and the catalytic subunit LET-92 (orange). The Arginine substituting the Tryptophane in position 140, in the *sur-6*ts mutant, is highlighted in red. This residue is located at the interface between SUR-6 and the scaffold PAA-1 subunit and may destabilize the entire complex. Two different orientations and zoomed regions of the PP2A-B55^{SUR-6} structure showing the position of the Arginine 140 in red are presented.

 © The Author(s)
